# Structure of Self-Initiated Photopolymerized Films: A Comparison of Models

**DOI:** 10.1021/acs.langmuir.2c02396

**Published:** 2022-11-03

**Authors:** Béla Nagy, Tobias Ekblad, Giovanna Fragneto, Thomas Ederth

**Affiliations:** † Division of Biophysics and Bioengineering, Department of Physics, Chemistry and Biology, Linköping University, SE-581 83Linköping, Sweden; ‡ Institut Laue-Langevin, 71 avenue des Martyrs, BP 156, 38042Grenoble, France

## Abstract

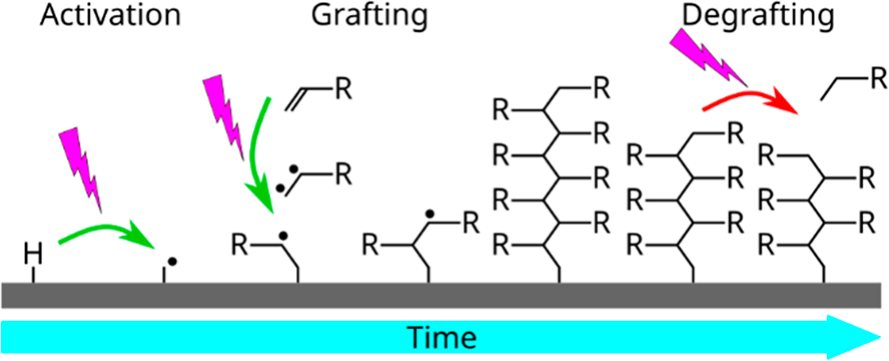

Self-initiated photografting and photopolymerization (SI-PGP) uses UV illumination to graft polymers to surfaces without additional photoinitiators using the monomers as initiators, “inimers”. A wider use of this method is obstructed by a lack of understanding of the resulting, presumably heterogeneous, polymer structure and of the parallel degradation under continuous UV illumination. We have used neutron reflectometry to investigate the structure of hydrated SI-PGP-prepared poly(HEMA-*co*-PEG_10_MA) (poly(2-hydroxyethyl methacrylate-*co-*(ethylene glycol)_10_ methacrylate)) films and compared parabolic, sigmoidal, and Gaussian models for the polymer volume fraction distributions. Results from fitting these models to the data suggest that either model can be used to approximate the volume fraction profile to similar accuracy. In addition, a second layer of deuterated poly(methacrylic acid) (poly(dMAA)) was grafted over the existing poly(HEMA-*co*-PEG_10_MA) layer, and the resulting double-grafted films were also studied by neutron reflectometry to shed light on the UV-polymerization process and the inevitable UV-induced degradation which competes with the grafting.

## Introduction

Polymer brushes^1,2^ are polymer chains covalently anchored to a surface at one end to form a dense layer that forces chains to stretch and are widely used to improve surface properties for lubrication,^3^ antifouling,^3,4^ and biosensing^5^ and in medicine.^6^ Brushes can be prepared either by grafting polymer chains directly to a surface (“grafting to”) or by growing the polymer chains from initiators attached to the surface (“grafting from”), where the latter is usually considered to offer better control of grafting density and brush thickness.^7^ Two widely used methods to graft brushes from surfaces are surface-initiated atom transfer radical polymerization (SI-ATRP) and surface-initiated reversible addition–fragmentation chain transfer (SI-RAFT). However, these are not ideal methods. The major disadvantages of conventional SI-ATRP are that it requires an oxygen-free environment, that it uses halogenated metal catalysts which may be harmful for some applications or the environment, and the inability to reuse the polymerization solution. SI-RAFT generally does not produce polymer brushes as thick as other surface-initiated controlled radical polymerization (CRP) techniques. Typical thicknesses are <30 nm using SI-RAFT. Also, the RAFT agent is often expensive or not commercially available; therefore, multiple-step syntheses may be needed.

Self-initiated photografting and photopolymerization (SI-PGP) is a method that uses UV light to polymerize monomers without additional photoinitiators but using initiator monomers (“inimers”). It was first reported using styrene monomers^8^ and later with acrylic or methacrylic monomers,^9^ poly(ether ether ketone),^10^ and poly(disulfide) oligomers.^11^ This method can be used to graft polymers onto surfaces that are difficult to functionalize, like polyethylene,^12,13^ graphene,^14^ carbon nanotubes,^15,16^ or hexagonal boron nitride.^17^ Recent applications include grafting onto cellulose nanocrystals^18^ during the synthesis of metal–organic frameworks^19,20^ and to improve the device capabilities of perovskite quantum wells.^21^ Being a light-induced process, the preparation of patterned films^22−25^ and gradients^26−28^ is straightforward. In the past, we have studied physicochemical properties^29^ and the antifouling performance of poly(HEMA-*co*-PEG_10_MA) (poly(2-hydroxyethylene methacrylate-*co*-poly(ethylene glycol)_10_ methacrylate)) films prepared by SI-PGP.^30,31^ In addition, the effects of charge imbalance on polymer hydration and swelling,^32^ their potential use for antifouling,^33^ and the adhesion of proteins on gradients of oppositely charged polyelectrolytes, prepared by the same method, were investigated.^28,34,35^

Unlike the SI-ATRP and SI-RAFT reactions, which result in linear polymers, SI-PGP is a less defined process. The initiator-free UV-induced polymerization can proceed via different reactions: by photolysis and radical formation of C=C or C=O bonds or via formation of biradicals at the vinyl group.^8^ This means that the formed polymer is likely heterogeneous, possibly with some degree of branching and/or cross-linking. End-grafted polymer chains in the brush limit have previously been described using scaling theory^36^ and self-consistent field theory^37,38^ along with Monte Carlo^39^ and molecular dynamics simulations.^40^ Experimental work on tethered diblock copolymers at the air/liquid interface was performed in good,^41^ theta,^42^ and poor^43^ solvent conditions to investigate how the brush height depends on molecular weight and grafting density and to determine the polymer volume fraction profiles. These parameters largely determine the performance of the brushes in applications and are thus of central interest. However, there is no accepted model describing, for example, chain segment density distributions in films created by the SI-PGP method. In parallel with the UV polymerization, there is also a continuous UV-induced degradation, which will affect the resulting structure. UV-induced degradation of plastics under natural or artificial light has been studied intensely for a very long time,^44,45^ and studies pertinent to SI-PGP-prepared polymers are also available.^30^ Determining the chain-end profiles and polymer volume fraction distributions for SI-PGP-prepared polymer layers might improve the understanding of the UV-grafting process, allow for better control of film formation, and may lead to further applications of such polymers. Compared to many CRP methods, the SI-PGP method is simple and inexpensive, in that no controlled atmosphere is required, no chemicals are needed beyond the monomers and the solvent (avoiding potentially toxic halide initiators, transition-metal ligands, or chain transfer agents), and the process is fast and might allow for a 100–1000-fold reduction in the amount of used monomers.^33^ This means that the potential benefits of the SI-PGP process for engineering applications could be considerable and that efforts to better understand the process are justified.

Neutron reflectometry is a well-suited tool for investigating thin films on planar surfaces. Neutrons striking an interface at a shallow angle, θ, may be reflected, and reflections from different layers at the interface result in an interference pattern, which can be recorded as a function of the momentum transfer normal to the surface *Q*_*z*_ = 4π sin θ/λ, where λ is the wavelength of the incident beam. By fitting a model to this pattern, the refractive index depth profile of the sample can be determined. Neutrons interact with the nuclei of the target atoms, which makes them sensitive to isotope substitution. In soft matter studies, parts of the sample are commonly labeled by using deuterated chemicals as a means of varying the contrast within the sample.^46^ Furthermore, the polymer volume fraction depth profiles can be determined by measuring the same sample using different mixtures of deuterated and hydrogenated solvent and simultaneously fitting the recorded reflectograms to the same model. Neutron reflectometry data are generally ill-conditioned for fitting due to the loss of phase information during the detection of the reflected neutron wave.^47^ To increase the confidence of the model fit in the case of hydrated samples, multiple reflectograms with different mixtures of D_2_O and H_2_O are measured and fit simultaneously beyond the necessary two compositions that are required to determine the solvent content of the layers. To further increase this confidence, the reflectograms of dry polymer samples are also fit together with the hydrated data, providing constraints on the total amount of polymer.

In this paper, neutron reflectograms obtained from poly(HEMA-*co*-PEG_10_MA) layers, grafted onto silicon and gold substrates via the SI-PGP method, are modeled to determine the polymer volume fraction profiles of the films. Copolymers prepared from mixtures of HEMA and PEG_10_MA result in better reproducibility and antifouling performance and are easier to prepare to large thicknesses than films prepared from either homopolymer.^30^ The exact ratio of the two monomers is a compromise between growth rate, thickness reproducibility, UV stability, and antifouling performance, but poly(HEMA-*co*-PEG_10_MA) layers prepared from 1:1 mixtures of HEMA and PEG_10_MA have been used in several previous studies,^22,23,26,29−31,48,49^ which motivated our preference for this mixture also in this study. The silicon and gold substrates were selected based on previous observations that grafting rates differ considerably between them^29^ and also because they are commonly used as substrates in biosensor applications, where SI-PGP films have been used to prevent nonspecific protein adsorption.^23^ To estimate possible cross-linking and the effects of successive grafting, an additional layer of deuterated methacrylic acid (MAA) was grafted on the samples with poly(HEMA-*co*-PEG_10_MA) coatings, which were also investigated with reflectometry.

## Materials and Methods

### Chemicals and Materials

All water was Type 1 ultrapure water (18.2 MΩ·cm resistivity). Ammonia and hydrogen peroxide were obtained from VWR (AnalR Normapure), ethanol (99.5%) from Solveco AB, Sweden, glacial acetic acid from Merck, and γ-methacryloxypropyltrimethoxysilane (MPS, sold as PlusOne Bind-Silane) was purchased from GE Healthcare Life Sciences, Sweden (now Cytiva). Deuterated methacrylic acid (dMAA) was purchased from Polymer Source Inc. (Montreal, Canada). 2-Hydroxyethyl methacrylate (HEMA) and poly(ethylene glycol) methacrylate (PEG_10_MA) with an average molecular weight of 500 g/mol were bought from Sigma-Aldrich. 16-Thiohexadecanol was obtained from Biacore AB, Sweden (now Cytiva).

### Sample Preparation

Two samples were grafted onto polished silicon surfaces, labeled Si1 and Si2. For this purpose, the polished (111) faces of 50 × 50 × 10 mm^3^ undoped Si blocks with native oxide layers were used. Before polymer grafting, the surfaces were cleaned using the TL-1 procedure (aka RCA SC-1, sample immersed for 5 min in a 5:1:1 mixture of H_2_O, 25% NH_3_, and 30% H_2_O_2_ at 85 °C), and a silane layer was deposited to serve as an organic layer to graft the polymer onto. For this, the blocks were submerged in a 1:1 solution of ethanol and water containing 0.4% MPS and 0.05% glacial acetic acid for 5 min and then baked at 115 °C for 10 min. The blocks were ultrasonicated in ethanol for 10 s to remove unbound silanes, further rinsed with ethanol, and dried.

Two other samples, labeled Au1 and Au2, were prepared on gold-coated surfaces. The gold surfaces were prepared by depositing first a 1.5 nm Ti adhesion layer and then 15 nm Au onto the TL-1 cleaned, polished surfaces of two other Si blocks from the same batch as above, using an electron-beam UHV evaporation system (Balzers UMS500P). Evaporation rates were set to 0.1 and 0.5 nm/s for Ti and Au, respectively. The base pressure was typically below 5 × 10^–9^ Torr before evaporation started, and the pressure during the gold evaporation step was ≤5 × 10^–8^ Torr. The coated blocks were then stored in sealed containers until further use. Before polymer coating, these blocks were TL1-cleaned again and immersed in a 1 mM solution of 16-thiohexadecanol in ethanol overnight, whereafter they were sonicated in ethanol for 2 min to remove physisorbed thiols, rinsed with ethanol, and dried.

The polymer coatings were prepared using the SI-PGP method onto the silanized or thiolated substrates, respectively, and the two types of substrates were treated identically from this point (except for the polymerization times; see below). The first monomer solution consisted of 120 mM HEMA and 120 mM PEG_10_MA dissolved in water. The second was prepared by dissolving 1% w/w (109 mM) dMAA in phosphate-buffered saline (PBS) with a pH of 7.4. No initiator was added, and the monomers were used without purification. The polymerization process and the reactor setup are briefly described in the following but have been described in detail elsewhere.^31^ 130 μL of monomer solution was sandwiched between a UV-transparent quartz disc and the substrate by placing the liquid on the substrate and gently putting the quartz plate on top of the applied monomer solution. The sandwich was then placed at a fixed distance (45 mm), for a given time, under a UV lamp with the main emission peak at 254 nm (Philips TUV PL-L, 18 W). The different monomers were grafted sequentially, starting with the hydrogenated monomers. The grafting times for the individual samples are displayed in Table 1. The grafting times were chosen to be longer for the silicon substrates to account for the faster growth of polymers on gold substrates.^29^ It has been established in previous work that the resulting thickness varies nonlinearly with time, as monomer depletion and UV degradation continually decrease the growth rate.^30^

**Table 1:** Grafting Times for the Two Types of Monomer Solution for Each Individual Sample

sample	HEMA-*co*-PEG_10_MA	dMAA
Si1	3 min	4 min
Si2	4 min 30 s	4 min
Au1	1 min 30 s	
Au2	1 min	1 min 45 s

### Neutron Reflectometry

The neutron reflectometry measurements were performed at the D17 reflectometer^50^ at the Institut Laue-Langevin (ILL, Grenoble, France). For the silicon surfaces, reflectograms were recorded both before and after the deposition of the second layer, while on the Au2 surface, only after the completion of both depositions. The blocks were mounted onto the reflectometer in a liquid flow cell with the neutrons reaching the surface through the Si blocks in all measurements. Reflectograms were recorded using different mixtures of D_2_O and H_2_O as the solvent, as well as in the dry state, when the cell was purged with dry N_2_ gas. Each sample was measured in three or four solvent contrasts (further details are provided in the Supporting Information, Table S1). The contrasts were labeled as D_2_O and H_2_O for the pure isotopes and CMAu, CM4, and CMSi for mixtures with scattering length densities of 4.5 × 10^–6^, 4.0 × 10^–6^, and 2.07 × 10^–6^ Å^–2^, respectively. The CMAu and CMSi values were selected as their refractive indices are equal to those of Au and Si, respectively. By minimizing the contrast between the substrate and the solvent, the scattering from the polymer layer becomes the dominant source of the reflected signal. The CM4 contrast, as an additional contrast, was chosen because it is between the measured D_2_O and CMSi contrasts. Measuring multiple contrasts increases the precision in the determination of the volume fraction profiles. Further details about optimizing reflectometry measurements can be found in the work of Durant et al.^51^ The reflectometer was set to time-of-flight mode, and the beam height (with a vertically mounted sample) was set to 38 mm. Slits S1 (at the guide exit) and S4 (in front of the detector) were fixed to 4 mm widths. The samples were measured at 0.7° and 3° angles of incidence with slits S2 (at the exit of the collimation guide) fixed to 0.5 and 3.4 mm and S3 (immediately after the sample) to 0.4 and 1.7 mm, respectively, and with the sample placed between slits S2 and S3. The measuring times were 15–120 min for the different contrasts (see Table S1 for details).

### Modeling

All the reflectograms recorded at a given grafting stage on each sample were fit simultaneously using the GenX program.^52^ The fits were minimized with respect to a logarithmic figure of merit (FoM), and the presented error bars correspond to a 5% increase in the FoM. Hydration in the layers were considered by calculating the scattering length density (SLD) of the *n*th layer according to the equation
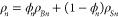
1where ρ_*n*_ is the SLD of the *n*th layer, ρ_*Bn*_ is the SLD of the nonaqueous material in the layer, ρ_*Sn*_ is the SLD of the water, and ϕ_*n*_ is the volume fraction of the material. Because the solvent contrast was varied using different mixtures of D_2_O and H_2_O, it is the volume fraction of the solvent which is determined in the experiments. We refer to the remaining volume fraction as the nonaqueous material in the sample. When modeling the reflectograms of the dry samples, the SLD of the solvent (air) was constrained to 0 Å^–2^. For thin layers, roughness values comparable to or greater than the layer thickness often lead to unphysical solutions. To avoid such results in the case of layers with thickness *d* < 50 Å, the roughness values on both interfaces were constrained to be identical.^53^

The silicon substrates (samples Si1 and Si2) were modeled using a native oxide layer and a silane layer on top of a silicon surface. The porosity of the native oxide is represented by the ϕ_SiO_2__ volume fraction parameter, constrained between 1 and 0.5. Also, the SLD ρ_SiO_2__ was set to 3.48 × 10^–6^ Å^–2^, and ρ_Si_ was fixed to 2.07 × 10^–6^ Å^–2^. The gold-coated substrates (samples Au1 and Au2) were modeled with a titanium adhesion layer, a gold layer, and a thioalkyl layer on top of a silicon substrate, also these with a native oxide layer. During the fitting, the SLD parameters ρ_Si_, ρ_Ti_, ρ_Au_, and ρ_Thiol_ were set to 2.07 × 10^–6^, –1.92 × 10^–6^, 4.5 × 10^–6^, and –0.5 × 10^–6^ Å^–2^, respectively. Self-assembled long-chain alkylthiol monolayers on gold surfaces form crystalline layers with few defects. To represent this, the parameter ϕ_Thiol_ was constrained between 1 and 0.9.

The dry hydrogenated films were modeled with the polymer as a single layer with ϕ_dry_ = 1 on top of an interface layer (see below). Because dry layers collapse, with only residual hydration, the dry and wet thickness parameters were decoupled during fitting. The hydrated polymer films were modeled by slicing the SLD profiles into 1 Å thick layers, keeping ρ_Poly_ a constant value restricted between the nominal values of the two components (ρ_HEMA_ = 0.99 × 10^–6^ Å^–2^ and ρ_PEGMA_ = 0.72 × 10^–6^ Å^–2^). To ensure a continuous transition between the SLD of the hydrated profile and the substrate, an interface layer was created with a thickness of 3σ_sub_, an SLD of ρ_Poly_, and a volume fraction equivalent to ϕ_Poly_(*z* = 0), where σ_sub_ is the roughness of the top layer of the substrate. When calculating the polymer volume fraction profiles, additional constraints were introduced for each model by limiting the total number of monomers to that calculated from the reflectograms obtained on the dry samples. These constraints are detailed in the Supporting Information. To investigate the structure of the film, three different polymer volume fraction profiles were fit to the data.

Parabolic volume fraction profiles are used to describe polymer brushes, where the conformation of the individual chains is influenced by the neighboring chains, resulting in stretching.^1^ To allow for a nonideal chain, the stretched exponential parabolic model^41^ was used in this study. The equation describing this profile is
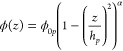
2where ϕ_0*p*_ is the polymer volume fraction near the interface, *h*_*p*_ is the height of the brush, and the exponent α describes the shape of the parabolic brush.

Collapsed polymer layers are modeled with functions that are used in approximating the unit step function. In other works,^54^ the tanh(*z*) function was used; here we use a single layer with sigmoidal roughness that is described as
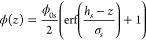
3ϕ_0*s*_ is the polymer volume fraction near the interface, *h*_*s*_ is the thickness of the layer, and σ_*s*_ is the roughness of the layer. To account for the tail of the sigmoidal profile stretching beyond *h*_*s*_, the splicing was done on a *h*_*s*_ + 3σ_*s*_ thick layer. The polymer volume fraction profile of polyelectrolyte brushes in the osmotic regime is described using a Gaussian profile as^55^
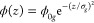
4where ϕ_0*g*_ is the polymer volume fraction near the interface and σ_*g*_ is the characteristic length of the profile. In this model the slicing was done on a *h*_*g*_ = 3σ_*g*_ thick layer. The “osmotic regime” refers to strongly charged polyelectrolyte brushes at low ionic strength, with extended chains and where the brush height is relatively independent of the grafting density and the ionic strength of the solution. For the parabolic and sigmoidal models describing the hydrated structure of the sample, applying the constraints required for simultaneous fitting is not trivial. Although a method based on Lagrange multipliers was used in previous studies,^56^ we use numerical calculations implemented in a Python script within the GenX program^52^ to constrain the fit parameters; details of these procedures, including the code, are included in the Supporting Information.

To assess the swelling of the layers, we defined the hydrated layer thickness using the expression
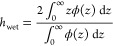
5The formulas for calculating the first moment of the volume fraction profiles are shown in the Supporting Information (Table S3). The swelling of the layer can then be calculated as Σ = *h*_wet_/*h*_dry_.

The monomer ratios in the films were determined using the following equation:
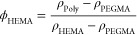
where ϕ_HEMA_ is the volume fraction of the HEMA monomer. The surface coverage values (Θ) for the Gaussian models were calculated according to the following formula
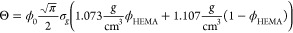
where 1.073 and 1.107 are the densities of HEMA and PEG_10_MA, respectively. The SLD profiles of dry films after grafting of a second layer from deuterated monomers were approximated with two layers and the hydrated films with four layers. The SLD values have been constrained between the nominal values of PEG_10_MA and dMAA (ρ_dMAA_ = 5.53 × 10^–6^ Å^–2^). Because the fits are only an approximation of the real profiles, the layers in the fit do not necessarily represent an actual stratified film; thus, the remaining parameters are not constrained to *a priori* specified physically realistic values. Because UV radiation is known to modify the structure of oxide layers on silicon wafers^57,58^ (see also Table S2 and related comments on this in the Supporting Information), the substrate parameters from the previous fit (after the first polymer layers) were not directly adapted for these fits, but they were determined once again.

## Results and Discussion

### Single-Grafted Layers

**Figure 1:**
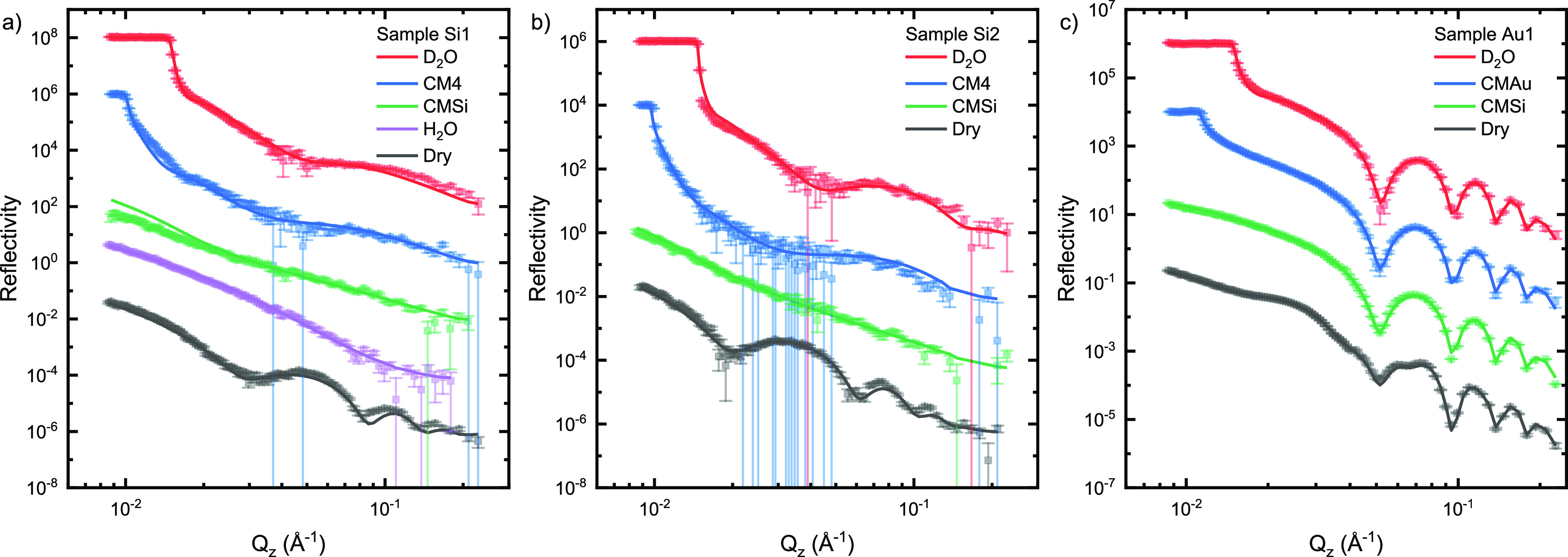
Neutron reflectometry data measured on hydrogenated films (dots) after one grafting and the resulting fits from the modeling (lines), using the Gaussian fit model for samples Si1 (a), Si2 (b), and Au1 (c). The data for dry samples (black) are correctly positioned relative to the vertical axis, and subsequent data sets have been scaled ×100 relative to the previous data set for clarity. For the hydrated measurement the contrasts were labeled as D_2_O and H_2_O for the pure isotopes and CMAu, CM4, and CMSi for mixtures with scattering length densities of 4.5 × 10^–6^, 4.0 × 10^–6^, and 2.07 × 10^–6^ Å^–2^, respectively.

Neutron reflectometry data from the poly(HEMA-*co*-PEG_10_MA) films are presented in Figure 1. To determine the polymer volume fraction model best describing the reflectometry data, the figures of merit for the fits to the different models are compared. For this the χ^2^ statistic (χ^2^) is frequently used. However, reflectometry data are ill-conditioned for fitting based on the χ^2^ figure of merit (FoM). Because the data are spanning several orders of magnitude in value, the χ^2^ FoM tends to favor the low-*Q*_*z*_ region of the reflectograms. To improve the fitting in other *Q*_*z*_ regions, the logarithm of the data and the model curve can be compared instead, resulting in a logarithmic FoM. This method favors the middle *Q*_*z*_ region of the reflectograms where most of the information about the shape of the SLD profile is contained. For this study we opted to use the logarithmic FoM. Model curves resulting from the fitting using both FoMs are shown in the Supporting Information (Figures S5, S7, and S9). The log FoM values for the different models are shown in Table S4. The overall much lower FoMs for the Au1 data are caused by the gold layer dominating the scattering. The values show only minor differences between the different models within each sample, implying that the models fit the data equally well. A comparison of the resulting volume fraction profiles (shown in Figures S4C, S6C, and S8C) indicates that the models are describing overall similar profiles. In Figure S4C, the sigmoidal profile differs from the other two, while in Figure S6C, the parabolic profile differs from the other two. In both these cases, it is clear from Figures S4A and S6A that the deviating volume fraction profiles are also associated with unsatisfactory fits to the data, whereas Gaussian profiles represent the experimental data well in all three cases. The deviating fits could be an effect of the fitting procedure being trapped in a local minimum or reflect a genuine inconsistency between the model and the data.

However, evaluation of the parameters describing the polymer layers, for the different models, shown in Table 2, reveal that only the fit to the Gaussian model resulted in parameters within the ranges expected from the models. Calculations using self-consistent field theory in the asymptotic limit predict that tightly packed polymer brushes have an α value of 1/2 in the parabolic model.^59,60^ Experimental results^42^ confirmed by simulations^61^ found that in practical cases the exponent is larger, and it approaches α = 2 from above as the overlap of the chains increases. In our models the α values were calculated from the polymer amount constraint, and all values are far greater than the values expected from the cited works. Thus, in our case this suggests either that the cause of the stretching observed on the samples is something else than just the excluded volume effect of the neighboring polymer chains or that the chain length distribution is different.

**Table 2:** Parameters Describing the Polymer Layer from the Parabolic, Sigmoidal, and Gaussian Models for Samples Si1, Si2, and Au1^a^

sample	Si1	Si2	Au1
parabolic model			
ρ_*p*_ (×10^–6^ Å^–2^)	0.77 ± 0.12	0.88 ± 0.07	0.95 ± 0.09
ϕ_0*p*_	0.44 ± 0.04	0.50 ± 0.03	0.86 ± 0.03
α^b^	13 ± 11	60 ± 20	30 ± 20
*h*_*p*_ (Å)	800 ± 300	2000 ± 200	1600 ± 700
sigmoidal model			
ρ_*s*_ (×10^–6^ Å^–2^)	0.75 ± 0.10	0.90 ± 0.07	0.94 ± 0.10
ϕ_0*s*_	0.37 ± 0.05	0.55 ± 0.04	0.93 ± 0.05
σ_*s*_ (Å)	240 ± 170	206 ± 15	220 ± 30
*h*_*s*_ (Å)^b^	290 ± 90	190 ± 30	220 ± 30
Gaussian model			
ρ_*g*_ (×10^–6^ Å^–2^)	0.77 ± 0.11	0.88 ± 0.07	0.95 ± 0.09
ϕ_0*g*_	0.44 ± 0.04	0.48 ± 0.02	0.86 ± 0.03
σ_*g*_ (Å)^b^	210 ± 40	260 ± 20	280 ± 19
*h*_*g*_ (Å)^b^	620	770	839

a The displayed errors are estimates calculated from a ±5% increase in the FOM. In the table, ρ represents the SLD of the polymer, ϕ_0_ is the volume fraction at the solid surface, and *h* is the brush height for the respective model. α is the stretching exponent for the parabolic profile, σ_*s*_ is the roughness of the sigmoidal layer, and σ**_*g*_** is the characteristic length of the Gaussian profile.

b Denotes calculated parameters.

Sigmoidal profiles describe a collapsed layer. When the interfacial roughness σ_*s*_ is comparable to the brush height *h*_*s*_, the difference in the parameter ϕ_0_ and the actual value of ϕ(0) obtained from the model is very large. When σ_*s*_ ≤ 2*h*_*s*_/3, this difference is <1.69%. The sigmoidal fits in cases where σ_*s*_ > 2*h*_*s*_/3 result in highly stretched layers without any collapsed constant volume fraction regions. Further discussion about the effects of large σ_*s*_ values compared to *h*_*s*_ is found in the Supporting Information (page S4). On the basis of these arguments, we accept the Gaussian model as that which is best suited to describe our samples. While the Gaussian model was developed for polyelectrolytes, where charge–charge interactions cause stretching of the polymer chains, charges are not present in our polymers, and the mechanism causing stretching needs to be found elsewhere. However, charges are not explicit in the model either, and as such the model is indifferent to the cause of the stretching; thus, there is no reason to object to such a description on grounds of principle. The curves from the Gaussian model fits (using log FoM) are presented in Figure 1, and the corresponding parameters are given in Table 3.

**Table 3:** Fit Parameters for the Hydrogenated Layers Modeled with a Gaussian Polymer Volume Fraction Profile^a^

parameter	Si1	Si2	parameter	Au1
*d*_SiO_2__ (Å)	18 ± 2	14.6 ± 1.4	*d*_SiO_2__ (Å)	5.0 ± 1.8
σ_SiO_2__ (Å)	3.0 ± 1.9	5 ± 2	σ_SiO_2__ (Å)	5.9 ± 0.8
ϕ_SiO_2__	0.60 ± 0.06	0.64 ± 0.07	*d*_Ti_ (Å)	10.1 ± 0.6
			*d*_Au_ (Å)	143.1 ± 0.7
*d*_Silane_ (Å)	27 ± 5	42 ± 5	*d*_Thiol_ (Å)	18.7 ± 1.3
ϕ_Silane_	0.64 ± 0.05	0.66 ± 0.02	ϕ_Thiol_	0.97 ± 0.03
ρ_Silane_ (×10^–6^ Å^–2^)	1.10 ± 0.20	1.21 ± 0.09	ρ_Thiol_ (×10^–6^ Å^–2^)	–0.50 ± 0.13
σ_Silane_ (Å)	17 ± 8	3 ± 4	σ_Thiol_ (Å)	3.0 ± 0.8
ρ_*g*_ (×10^–6^ Å^–2^)	0.77 ± 0.11	0.88 ± 0.07	ρ_*g*_ (×10^–6^ Å^–2^)	0.95 ± 0.09
ϕ_0*g*_	0.44 ± 0.04	0.48 ± 0.02	ϕ_0*g*_	0.86 ± 0.03
σ_*g*_ (Å)^b^	210 ± 40	260 ± 20	σ_*g*_ (Å)^b^	280 ± 19
*h*_*g*_ (Å)^b^	620	770	*h*_*g*_ (Å)^b^	839
*h*_dry_ (Å)	80 ± 7	109 ± 6	*h*_dry_ (Å)	213 ± 7
σ_dry_ (Å)	3 ± 20	13 ± 10	σ_dry_ (Å)	6 ± 11
*h*_wet_ (Å)^b^	233	290	*h*_wet_ (Å)^b^	316
swelling^b^ (%)	291	266	swelling^b^ (%)	148
HEMA content (%)^b^	20 ± 40	60 ± 30	HEMA content (%)^b^	80 ± 30
surface coverage (ng/cm^2^)^b^	900 ± 190	1200 ± 110	surface coverage (ng/cm^2^)^b^	2300 ± 180

a The displayed errors are estimates calculated from a ±5% increase in the FOM. *d* is layer thickness, σ is interfacial roughness, ρ is SLD, ϕ is volume fraction, ϕ_0_ is volume fraction at the solid surface, and *h* is the brush height.

b Denotes calculated parameters.

**Figure 2:**
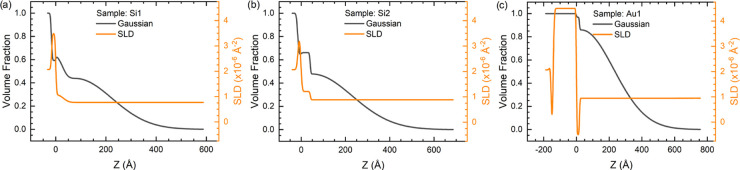
Volume fraction profiles (black) and SLD profiles (orange) of the nonaqueous components calculated from the model fits on the hydrogenated layers for samples Si1 (a), Si2 (b), and Au1 (c). The zero of the thickness (*Z*) is set to the interface of the SiO_2_/silane and gold/alkylthiol layers for the silicon (Si) and gold (Au) samples, respectively.

**Figure 3:**
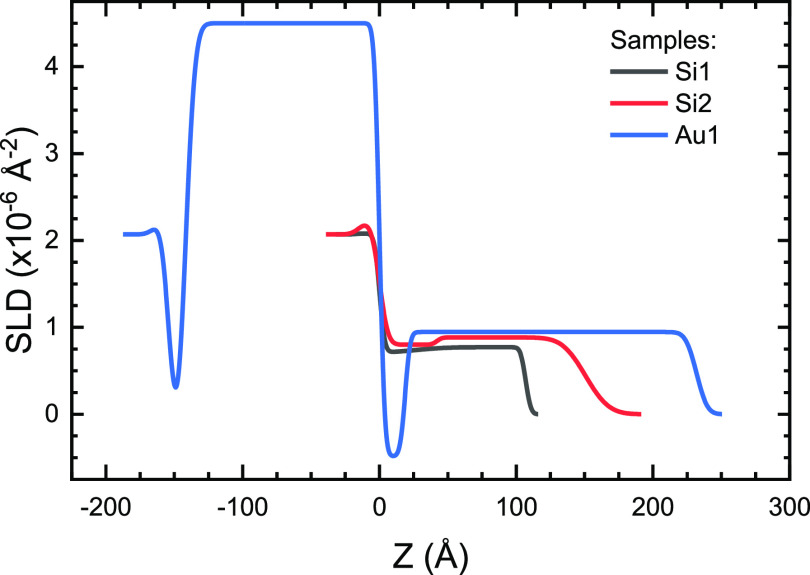
SLD profiles of the hydrogenated samples measured in the dry state. The zero of the thickness is set to the interface of the SiO_2_/silane and gold/alkylthiol layers for the silicon (Si) and gold (Au) samples, respectively.

The volume fractions and SLD profiles of the (nonaqueous components of the) samples calculated from the models are shown in Figure 2. The SLD profiles for the dry layers are presented in Figure 3. The model parameters for the fitted curves as well as SLD and polymer volume fraction profiles for all three models are presented in the Supporting Information (Figures S4–S9).

Gaussian polymer volume fraction profiles are used to describe polyelectrolyte brushes in the osmotic limit^55^ and brushes in poor solvent.^43^ In the case of polyelectrolytes, the chains are stretched by the osmotic pressure of the counterions even below the θ point, but the electrostatic interactions between the charged monomers are screened. A detailed discussion and calculation may be found in Zhulina et al.^55^ In the present case the monomers do not contain charged groups, and infrared spectroscopy on identically prepared samples show no indication of ionizable residues in the films.^29^ However, recent work suggests that hydronium ions bound to PEG chains can form a so-called supra-polyelectrolyte, where PEG behaves like a pH-dependent polyelectrolyte rather than a conventional neutral polymer in an aqueous solution.^62^ This could potentially explain a polyelectrolyte-like behavior, but further data are needed to support such a hypothesis; leaving this—at this stage somewhat speculative—hypothesis aside, the behavior could be attributed to the entropic contribution of water molecules participating in hydrogen bonding with the side chains. Poly(ethylene glycol) is known for interacting strongly with water,^63,64^ resulting in strong hydration, which supports this hypothesis, but neutron reflectometry data from PEG brushes are usually modeled with parabolic profiles rather than Gaussian profiles.^37,65,66^ In poor solvent the polymer chains are collapsed by the unfavorable solvent–monomer interactions. As stated above, PEG interacts with water strongly, making this case unlikely. Furthermore, reported experimental polymer volume fraction profiles on tethered chains in poor solvent contain a constant volume fraction region near the substrate, which is missing from our results.

Another possible explanation for the suitability of Gaussian profiles could be stretching caused by overlapping of the neighboring PEG_10_MA side chains. Grafted polymer architectures with long side chains, called bottlebrushes, have received increased interest recently due to improvements in grafting methods and promising applications.^67,68^ Similar systems made using PEG_45_MA and HEMA and their interactions with SDS have been studied before,^69^ but neutron reflectometry studies were only performed on polyelectrolyte systems where HEMA was substituted with 2-(trimethylammonio)ethyl methacrylate chloride (METAC).^70^ However, scaling analysis combined with free energy calculations and molecular dynamics simulations of bottlebrushes with increasing grafting density resulted in increasing chain heights even at low and intermediate grafting densities.^71^ To assess the relevance of bottlebrush models for approximating the structure of SI-PGP polymers, the chain structure needs to be known, but determining the exact HEMA:PEG_10_MA monomer ratio in the current films is difficult due to lack of contrast between the monomers, which is represented in the large error values for the HEMA content of the films (Table 3). Calculating the weighted average results in 59 ± 16% HEMA content, which is near the HEMA concentration in the monomer solution used for the polymerization, indicating approximately one PEG_10_MA monomer for every other unit on the backbone.

The ϕ_0*g*_ values are 47.2 ± 1.6% for the polymers on Si substrates and 86 ± 3% for the one on Au substrate, and these near-substrate volume fractions can be approximated to grafting densities. For each sample, we also note that the ϕ_0_ values are very consistent across the three models. The ratio of these grafting densities is not unlike the ratio of the densities of the silane and thiol layers (see Table 3), and it seems reasonable to hypothesize that the densities of these organic layers influence the resulting polymer grafting densities. However, previous work showing effective SI-PGP poly(HEMA-*co*-PEG_10_MA) grafting on, for example, amorphous polyolefins^26^ or polystyrene^49^ indicate that end-terminating functional groups are not a requirement for grafting. This issue was also discussed in previous work on other SI-PGP-prepared polymers^33^ where we note that aliphatic hydrogens are very likely targets for hydrogen abstraction and radical formation and thus that grafting could occur on already grafted chains and is not limited to the end-groups of the silane or thiol layers. As a result, soon after grafting commences, there will be plenty of potential grafting sites on either surface type, reducing the influence of the density of the underlying organic monolayer. Although we cannot rule out an effect of the differences in the grafting densities of the silane and alkylthiol films on the obtained ϕ_0*g*_ values, we attribute this difference to the equilibrium between grafting and removal of monomers by the UV radiation. The reflectance for UV light is double from a silicon substrate compared to that from a gold layer.^29^ This increase in intensity near the surface promotes the degradation of the layer over silicon. Calculating the average grafting rates from the thicknesses of the dry samples in Table 3 and the grafting times in Table 1 yields the average growth rates 27 ± 2, 24.2 ± 1.3, and 142 ± 5 Å/min for samples Si1, Si2, and Au1, respectively. Although the growth rates are not constant, but decrease continuously with monomer depletion in the volume near the interface accessible through diffusion and UV degradation, and detailed comparisons for different polymerization times can be misleading, it is still clear from these averages that the ratio of the growth rates between the Si and Au samples are far from the ratio of the densities of the silane and thiol layers. It seems unlikely that the differences in grafting rates can be attributed to differences in the densities of the organic layers. Because the polymerization is progressing largely in bulk with attachment of chains to the surface, as a “grafting-to” process,^32^ the difference in the UV intensity also explains the different grafting speeds for the two surfaces. Studies performed on poly(HEMA-*co*-PEG_10_MA) brushes grown by surface-initiated atom transfer radical polymerization on gold substrates^72^ suggest a correlation between the crowding of the layers, swelling, and antifouling performance. Comparing the swelling and grafting in Figure S10, we can see a clear negative correlation between these in our samples as well, although data are limited.

### Double-Grafted Layers

**Figure 4:**
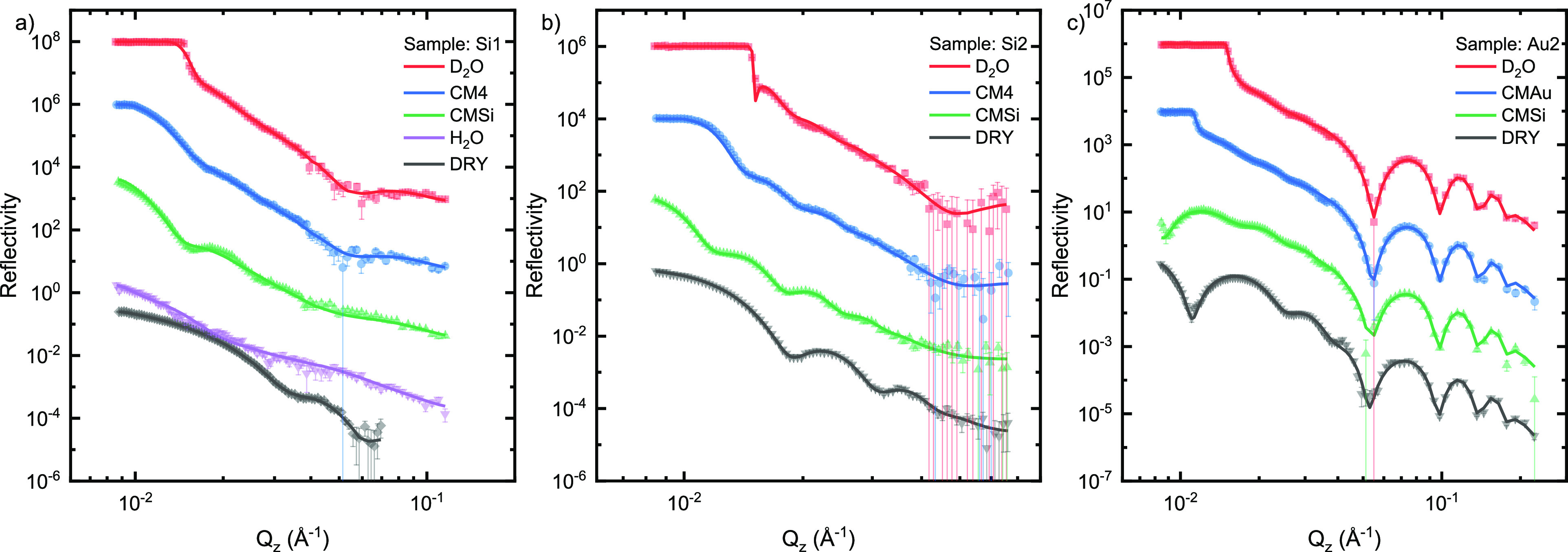
Neutron reflectometry data (dots) measured on double-grafted layers for samples Si1 (a), Si2 (b), and Au2 (c) with a deuterated MAA layer grafted after preparation of the hydrogenated films and the resulting fits from the modeling (lines). The data for the dry films (black) are correctly positioned relative to the vertical axis, and subsequent data sets have been scaled ×100 relative to the previous data set for clarity. For the hydrated measurements the contrasts were labeled as D_2_O and H_2_O for the pure isotopes and as CMAu, CM4, and CMSi for mixtures with scattering length densities of 4.5 × 10^–6^, 4.0 × 10^–6^, and 2.07 × 10^–6^ Å^–2^, respectively.

**Figure 5:**
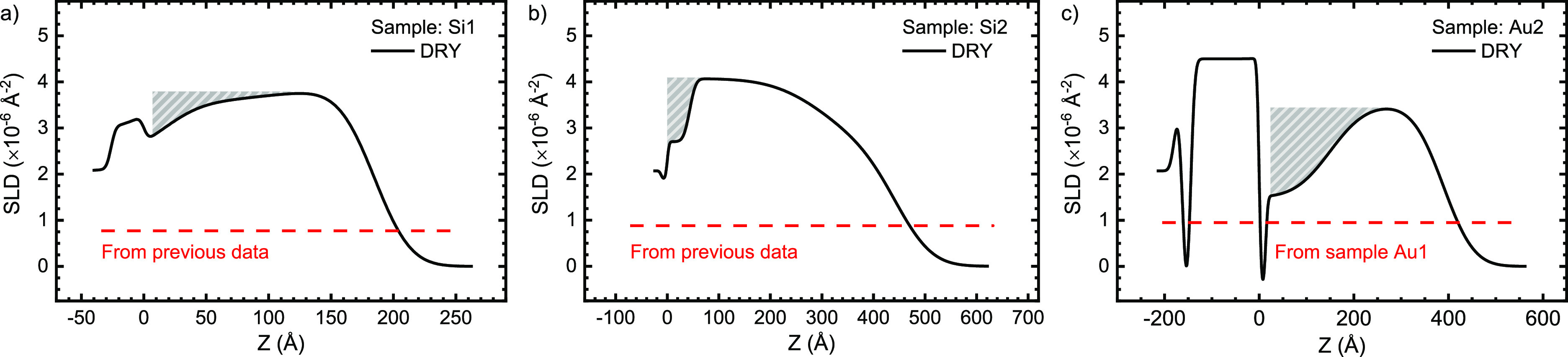
Scattering length density profiles of the deuterated samples (black) measured dry on samples Si1 (a), Si2 (b), and Au2 (c). For comparison, the scattering lengths of the hydrogenated layers are also shown (red dashed lines). For the Au2 sample the value from Au1 sample was used. The shaded area indicates the presence of hydrogenated material in the sample, that is, decreasing the SLD near the surface. The zero of the thickness is set to the interface of the SiO_2_/silane and gold/alkylthiol layers for the silicon (Si) and gold (Au) samples, respectively.

**Figure 6:**
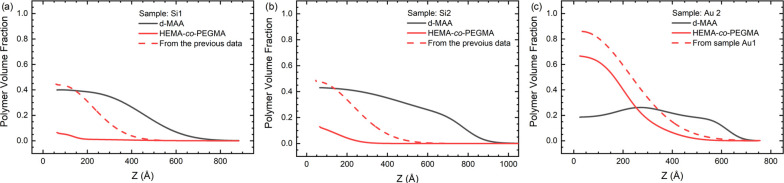
Volume fraction profiles of the deuterated (black) and hydrogenated (red) monomers in the hydrated double-grafted films on samples Si1 (a), Si2 (b), and Au2 (c). For comparison, the volume fractions of the hydrogenated monomer from the previous measurements (before the second grafting) are also displayed as dashed red lines.

To investigate the nature of grafting in SI-PGP, deuterated methacrylic acid (dMAA) was grafted onto the hydrogenated layers on samples Si1 and Si2 and onto a newly deposited layer on sample Au2. The recorded reflectograms and the results of the approximation fits are presented in Figure 4. The fit parameters are displayed in the Supporting Information (Table S8), the SLD profiles for the dry layers are shown in Figure 5, and the hydrated polymer volume fraction profiles are displayed in Figure 6.

Comparing the SLD profiles from the dry measurements to the ρ_*g*_ values obtained from the fits of the single-grafted layers (dashed red lines in Figure 5), we can see that the increase in SLD caused by the deuterated monomers is present even near the surface on all three samples, but significantly more so on Si1 and Si2. This suggests that the new monomers graft not only on top of the previous chains but also along their length and also directly onto the substrate. This is in accordance with our previous results regarding polyelectrolyte layers sequentially grafted with the same procedure.^32^ The low-SLD regions (marked by the shaded areas in Figure 5) near the substrate surfaces are signs of remaining hydrogenated material from the poly(HEMA-*co*-PEG_10_MA) layer. The precise determination of the monomer ratio in the films is not possible, as the maximum values in the profiles do not reach the nominal value of the dMAA monomer (5.53 × 10^–6^ Å^–2^). Because hydrogenated material outside the maximum SLD of the polymer films in Figure 5 cannot be distinguished from air, this also prevents us from assessing the influence of the surface roughness on the monomer ratio estimates. Thus, we can determine the roughnesses of the SLD profiles, but we do not know to what extent the decay of the SLD at distances greater than the SLD maximum is a result of interfacial roughness, or hydrogenated material, adding uncertainty to the determination of the polymer volume fraction profile. In addition to the hydrogenated polymer, the low SLD values near the substrates may be caused by trapped water in the layer or an inhomogeneous collapsed layer with significant voids trapped between. For the single-grafted hydrogenated layers, the SLD values for the dry and hydrated films were identical, suggesting very low water content and a homogeneous collapsed layer with little void volume ratio, and there is no reason to expect otherwise for these samples. The samples were dried with N_2_ gas for at least an hour before the dry measurements. The drying process was investigated using infrared spectroscopy on poly(MAA) and poly(HEMA-*co*-PEG_10_MA) films, both separately and sequentially grafted, by monitoring the −OH stretching band in the absorption spectrum, and after an initial change during the first ca. 5 min, no significant difference in the water content was found during 1 h of drying (see Figure S12). These results all indicate that the low SLD values near the substrates correspond to hydrogenated material and not water.

**Figure 7:**
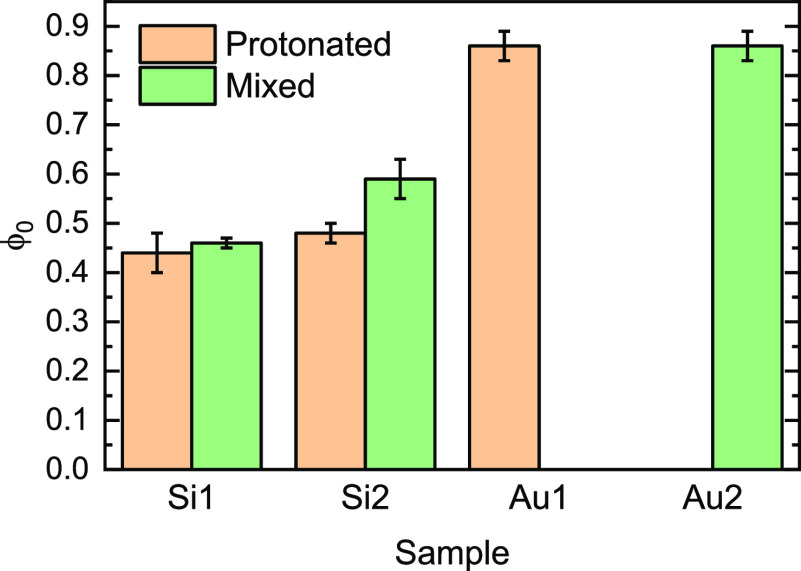
Polymer volume fractions near the interface before (amber) and after (green) the grafting of the deuterated layer.

From the fits of the reflectograms in Figure 4 one can calculate the volume fraction profiles of the hydrogenated and deuterated components using the SLD values obtained from the single-grafted hydrogenated fits. The results are shown in Figure 6. The results of the model fit show an increase in the deuterated monomer concentration near the end of the hydrogenated volume fraction profile in sample Au 2 (Figure 5C), which indicates direct grafting on top of the existing layer. The lack of this visible increase on the Si samples might be attributed to the near-complete removal of the previous layer. To assess the degrafting of the hydrogenated layer due to the UV illumination in the second grafting step, the polymer volume fractions from the previous measurements are displayed as red dashed lines in Figure 6. By integrating the hydrogenated polymer volume fraction profiles, we can determine that 9.2% and 13.1% of the original layer were remaining after the second grafting in the case of samples Si1 and Si2, respectively. In the case of the gold layer, by normalizing the polymer amount to the grafting time (between samples Au1 and Au2, because data for the first layer alone are not available for sample Au2), the estimate shows no degrafting with the deposition of the second layer. This is inaccurate as the polymer amount is not a linear function of the grafting time, but it does indicate significantly more retained hydrogenated polymer for sample Au2, possibly a result of the shorter grafting time and thus less degradation due to the additional UV illumination. Comparing the total polymer volume fraction values at the interface in Figure 7, we can see no major changes after depositing the deuterated layer. This suggests that the deuterated monomers graft to the surface only when the hydrogenated polymer was removed. This can mean that there is a finite number of anchoring points on the surface or that there is a steric barrier hindering the diffusion of new chains to the surface that is eased with the removal of polymer chains. Such a thickness self-limiting barrier is reported in the “grafting-trough” model of polymerization, where the polymerization takes place in the solution and the resulting chains diffuse to the surface and graft onto it through anchored monomers, from which the polymerization progresses further, forming essentially a combination of “grafting to” and “grafting from” growth.^73^ Because the alkylthiol layers on the Au substrates do not contain polymerizable groups, the “grafting-through” model is unlikely to be the case here. Also, within a “grafting-through” model, ϕ_0_ would be independent of the substrate but limited by the polymer layer, and the differences between the silicon and gold substrates shown in Figure 7 suggests that this is not the case. Thus, the data points in favor of a situation where the surface properties account for the observed differences between the silicon and gold substrates, either directly due to differences between the silane and the alkylthiol layers or indirectly via differences in reflectivity in the UV region, as discussed above.

### Implications for the SI-PGP Process

As explained in the Introduction, SI-PGP has many desired benefits for engineering applications, in that it is simple, inexpensive, and with potentially low environmental impact due to the small amount of required monomers, avoidance of toxic initiators, catalysts or chain transfer agents, and the use of water as a solvent. However, understanding the resulting film structure, the growth mechanism(s), and also how the simultaneous degradation by the UV illumination affects the polymer are all essential for wider use of this method. In previous work, we explored the polymerization mechanisms using oppositely charged monomers.^32^ The conditions for polymerization in such systems are very different from a system with nominally neutral monomers, in that charge–charge interactions could dominate the grafting process and have a strong influence on the resulting film structure. Indeed, it was found that the organization of the prepared films depend on electrostatic interactions, but a more general observation was that for sequential grafting of two different monomers growth of both polymers proceeded mainly via grafting of solution-polymerized fragments to the surface and also that the second layer is primarily grafted to the substrate and not as a continuation of the existing chains. These results were obtained on silicon substrates and agree with the current results for the Si1 and Si2 silicon substrates. The differences in this respect between films grafted on silicon and gold substrates are notable, and we consider the differences in reflectivity between the substrates in the relevant wavelength range the foremost hypothesis to account for this but must leave a definite resolution of this issue to future work.

The similarities between the FoM values obtained for the three tested models (Table S4), and also the visual similarities between the results (Figures S4, S6, and S8), would suggest that the choice of model for fitting our data is not a critical decision. To further illustrate this, calculated profiles for the same amount of polymer, for the three models (and with two different α parameters for the parabolic model) are included in Figure S11. From this figure it is clear that only minor differences in the predicted volume fraction or SLD profiles can be expected from the different models. We note that Kent et al. arrived at a similar conclusion in a very different polymer system (Langmuir monolayers of polydimethylsiloxane–polystyrene diblock copolymers), comparing four different models, and that their results were also in good agreement with self-consistent-field calculations of the polymer distribution.^41^ This is reassuring in that it indicates that selecting the “wrong” model might not be a huge problem but it also raises questions as to what can be considered reasonable parameter ranges for a physically realistic profile. Consider, for example, the comments on the expected values of the α parameter for the single-grafted layers mentioned previously. From the profiles in Figure S11, it is not obvious if, or why, an α parameter of 10 should be considered “unphysical” in any sense, and in the limit of large α parameters, the profile is approaching a Gaussian profile; accepting this as a physically viable profile implies that an upper limit of the α parameter appears unnecessary. Where model fits result in parameters outside of their expected range, this does not necessarily indicate an “unphysical” situation but rather that the particular interaction built into the model is not determining the structure. Thus, in these cases the interpretation of the fit results in physical terms must be done with care and take into account the underlying assumptions for the model parameters.

The random nature of the UV-grafting process, with uncontrolled radical formation propagating the polymerization, is expected to result in a heterogeneous polymer, including possibilities for cross-linking, which is also supported by some suggestions in the literature.^26^ Increasing cross-linking will give the films hydrogel character, but there are few structural studies of weakly or sparsely cross-linked surface-bound hydrogels available for comparison, and already moderate cross-linking results in relatively inflexible, homogeneously swelling slablike films. The work of Menzies et al., studying PEG-like plasma polymer films, is one of the few examples of surface-bound hydrogels studied by neutron reflectometry,^74^ showing merely a slab of relatively homogeneous composition and swelling upon hydration. Further examples in the literature include the hydration of poly(methyl methacrylate) films^75^ and the swelling of poly((diethylene glycol monomethyl ether methacrylate)-*co*-poly(ethylene glycol) methyl ether methacrylate) films^76^ in vapor, which were also modeling the polymer layer with a slab model.

In previous work using similarly prepared poly(HEMA-*co*-PEG_10_MA) films, Larsson and Liedberg have discussed the possibility of branching and cross-linking^26^ and, based on differences in the penetration of proteins into the polymer matrix, concluded that a “bushlike” structure is formed, with some degree of cross-linking, rather than a “brushlike” structure. From the obtained volume fraction profiles (see, for example, Figures 2 and 5), it is clear that there are density gradients along the surface normal in all samples, which could explain differences in penetration of differently sized proteins, whereas Larsson and Liedberg assumed that the films were homogeneous throughout the layer thicknesses. In addition, the successful fitting of our data to volume fraction distribution models developed for polymer brushes suggests that the degree of cross-linking is most likely very low and that the films largely retain the character of brushes. In addition, the agreement of our data with models with chain segment density distributions expected for stretched brushes also suggests that chain branching in the SI-PGP-prepared films is generally low.

## Conclusions

We have characterized SI-PGP-prepared poly(HEMA-*co*-PEG_10_MA) polymer thin films by neutron reflectometry, with a view to determining the resulting polymer chain segment density distributions under hydrated conditions. The investigation used both silicon and gold substrates not only because these are commonly used in biosensor applications where poly(HEMA-*co*-PEG_10_MA) films are used to minimize nonspecific protein adsorption but also because grafting under similar conditions results in different growth rates on these substrates.^29^ Understanding the structure of the polymer is not only essential for rational selection of materials and preparation conditions for coating applications but also of fundamental interest to understand the SI-PGP polymerization mechanism and how degradation under the continuous UV illumination affects the result. The reflectometry profiles were analyzed by fitting the data to three different models developed for polymer brushes—parabolic, sigmoidal, and Gaussian volume fraction profiles—and comparing the results. Results from the fitting indicate that the differences between these models are small, in that either could be adjusted to fit the data to similar fitting figures of merit, and hence that the choice of model in this case will not strongly affect the resulting volume fraction or SLD profiles. Overall, the results indicate that the films have a structure that is largely similar to that of end-grafted brushes, with little branching or cross-linking.

Grafting a second layer to samples with an existing layer aids in understanding the grafting process and can also clarify the impact of the UV illumination on the polymer. For this purpose, a second layer of deuterated poly(dMAA) was used. The deuteration allows separation of the distributions of the two monomers in the prepared films by neutron reflectometry. The results show that on silicon surfaces considerable degrafting of the first layer occurs, with significant amounts of the deuterated monomer near the substrate surface after the second polymerization. This is in contrast to the result for the gold substrates, where a much larger fraction of monomers from the second polymerization step remains on top of the first layer. We attribute this difference to the higher reflectance of silicon in the UV region, increasing the degradation rate, both slowing down the net growth rate, and also increasing the turnover of grafted chains in the second grafting step.

## Supporting Information Available

Acquisition details for the reflectometry experiments, effects of UV illumination on the native oxide layer on Si, procedures for constraining the amount of polymer in the models, figure-of-merit values for fits to the hydrogenated layers, comparisons of model fits using different models, deuterated layer parameters, graphs showing grafting density and the swelling of the layers, comparisons of polymer volume fraction profiles of different models, infrared spectroscopy data for hydration monitoring, Python code for model constraints (PDF)

## References

[ref1] Milner S. T. Polymer Brushes. Science 1991, 251 (4996), 905–914. 10.1126/science.251.4996.905. 17847384

[ref2] Chen W.-L.; Cordero R.; Tran H.; Ober C. K. 50th Anniversary Perspective: Polymer Brushes: Novel Surfaces for Future Materials. Macromolecules 2017, 50 (11), 4089–4113. 10.1021/acs.macromol.7b00450.

[ref3] Yang W.; Zhou F. Polymer brushes for antibiofouling and lubrication. Biosurface and Biotribology 2017, 3 (3), 97–114. 10.1016/j.bsbt.2017.10.001.

[ref4] Yang W. J.; Neoh K.-G.; Kang E.-T.; Teo S. L.-M.; Rittschof D. Polymer brush coatings for combating marine biofouling. Prog. Polym. Sci. 2014, 39 (5), 1017–1042. 10.1016/j.progpolymsci.2014.02.002.

[ref5] Badoux M.; Billing M.; Klok H.-A. Polymer brush interfaces for protein biosensing prepared by surface-initiated controlled radical polymerization. Polym. Chem. 2019, 10 (23), 2925–2951. 10.1039/C9PY00163H.

[ref6] Li D.; Xu L.; Wang J.; Gautrot J. E. Responsive Polymer Brush Design and Emerging Applications for Nanotheranostics. Adv. Healthcare Mater. 2021, 10 (5), 2000953 10.1002/adhm.202000953. PMC11468394 32893474

[ref7] Brittain W. J.; Minko S. A structural definition of polymer brushes. J. Polym. Sci., Part A: Polym. Chem. 2007, 45 (16), 3505–3512. 10.1002/pola.22180.

[ref8] Deng J.-P.; Yang W.-T.; Rånby B. Auto-Initiating Performance of Styrene on Surface Photografting Polymerization. Macromol. Rapid Commun. 2001, 22 (7), 535–538. 10.1002/1521-3927(20010401)22:7<535::AID-MARC535>3.0.CO;2-3.

[ref9] Wang H.; Brown H. R. Self-Initiated Photopolymerization and Photografting of Acrylic Monomers. Macromol. Rapid Commun. 2004, 25 (11), 1095–1099. 10.1002/marc.200400010.

[ref10] Kyomoto M.; Moro T.; Takatori Y.; Kawaguchi H.; Nakamura K.; Ishihara K. Self-initiated surface grafting with poly(2-methacryloyloxyethyl phosphorylcholine) on poly(ether-ether-ketone). Biomaterials 2010, 31 (6), 1017–1024. 10.1016/j.biomaterials.2009.10.055. 19906420

[ref11] Chemtob A.; Feillée N.; Vaulot C.; Ley C.; Le Nouen D. Self-Photopolymerization of Poly(disulfide) Oligomers. ACS Omega 2019, 4 (3), 5722–5730. 10.1021/acsomega.9b00021. 31459725 PMC6647947

[ref12] Wang H. Improving the Adhesion of Polyethylene by UV Grafting. J. Adhes. 2006, 82 (7), 731–745. 10.1080/00218460600775815.

[ref13] Han J.; Wang X.; Wang H. Superhydrophobic surface fabricated by bulk photografting of acrylic acid onto high-density polyethylene. J. Colloid Interface Sci. 2008, 326 (2), 360–365. 10.1016/j.jcis.2008.06.023. 18653198

[ref14] Steenackers M.; Gigler A. M.; Zhang N.; Deubel F.; Seifert M.; Hess L. H.; Lim C. H. Y. X.; Loh K. P.; Garrido J. A.; Jordan R.; Stutzmann M.; Sharp I. D. Polymer Brushes on Graphene. J. Am. Chem. Soc. 2011, 133 (27), 10490–10498. 10.1021/ja201052q. 21639111

[ref15] Toader M.; Schubel R.; Hartmann M.; Scharfenberg L.; Jordan R.; Mertig M.; Schulz S. E.; Gessner T.; Hermann S. Enhancement of carbon nanotube FET performance via direct synthesis of poly (sodium 4-styrenesulfonate) in the transistor channel. Chem. Phys. Lett. 2016, 661, 1–5. 10.1016/j.cplett.2016.07.049.

[ref16] Fresco-Cala B.; Carrasco-Correa E. J.; Cárdenas S.; Herrero-Martínez J. M. Carbon nanostructures incorporated on methacrylate monoliths for separation of small molecules by nano-liquid chromatography. Microchemical Journal 2018, 139, 222–229. 10.1016/j.microc.2018.03.003.

[ref17] Sheng W.; Amin I.; Neumann C.; Dong R.; Zhang T.; Wegener E.; Chen W.-L.; Förster P.; Tran H. Q.; Löffler M.; Winter A.; Rodriguez R. D.; Zschech E.; Ober C. K.; Feng X.; Turchanin A.; Jordan R. Polymer Brushes on Hexagonal Boron Nitride. Small 2019, 15 (19), 1805228 10.1002/smll.201805228. 30932320

[ref18] Hou L.; Bian H.; Wang Q.; Zhang N.; Liang Y.; Dong D. Direct functionalization of cellulose nanocrystals with polymer brushes via UV-induced polymerization: access to novel heterogeneous visible-light photocatalysts. Rsc Adv. 2016, 6 (58), 53062–53068. 10.1039/C6RA11403B.

[ref19] Hou L.; Wang L.; Zhang N.; Xie Z.; Dong D. Polymer brushes on metal-organic frameworks by UV-induced photopolymerization. Polym. Chem. 2016, 7 (37), 5828–5834. 10.1039/C6PY01008C.

[ref20] Hou L.; Zhou M.; Dong X.; Wang L.; Xie Z.; Dong D.; Zhang N. Controlled Growth of Metal-Organic Frameworks on Polymer Brushes. Chem.—Eur. J. 2017, 23 (54), 13337–13341. 10.1002/chem.201703827. 28816377

[ref21] Proppe A. H.; Wei M.; Chen B.; Quintero-Bermudez R.; Kelley S. O.; Sargent E. H. Photochemically Cross-Linked Quantum Well Ligands for 2D/3D Perovskite Photovoltaics with Improved Photovoltage and Stability. J. Am. Chem. Soc. 2019, 141 (36), 14180–14189. 10.1021/jacs.9b05083. 31422664

[ref22] Andersson O.; Larsson A.; Ekblad T.; Liedberg B. Gradient Hydrogel Matrix for Microarray and Biosensor Applications: An Imaging SPR Study. Biomacromolecules 2009, 10 (1), 142–148. 10.1021/bm801029b. 19067607

[ref23] Ekblad T.; Faxälv L.; Andersson O.; Wallmark N.; Larsson A.; Lindahl T. L.; Liedberg B. Patterned Hydrogels for Controlled Platelet Adhesion from Whole Blood and Plasma. Adv. Funct. Mater. 2010, 20 (15), 2396–2403. 10.1002/adfm.201000083.

[ref24] Cėpla V.; Rakickas T.; Stankevičienė G.; Mazėtytė-Godienė A.; Baradokė A.; Ruželė Ž.; Valiokas R. n. Photografting and Patterning of Poly(ethylene glycol) Methacrylate Hydrogel on Glass for Biochip Applications. ACS Appl. Mater. Interfaces 2020, 12 (29), 32233–32246. 10.1021/acsami.0c04085. 32438798

[ref25] Bai G.; Ma S.; Qie R.; Liu Z.; Shi Y.; Li C.; Wang R.; Guo X.; Zhou F.; Jia X. UV-Triggered Surface-Initiated Polymerization from Colorless Green Tea Polyphenol-Coated Surfaces. Macromol. Rapid Commun. 2016, 37 (15), 1256–1261. 10.1002/marc.201600065. 27272437

[ref26] Larsson A.; Liedberg B. Poly(ethylene glycol) Gradient for Biochip Development. Langmuir 2007, 23 (22), 11319–11325. 10.1021/la700729q. 17900155

[ref27] Steenackers M.; Küller A.; Stoycheva S.; Grunze M.; Jordan R. Structured and Gradient Polymer Brushes from Biphenylthiol Self-Assembled Monolayers by Self-Initiated Photografting and Photopolymerization (SIPGP). Langmuir 2009, 25 (4), 2225–2231. 10.1021/la803386c. 19140707

[ref28] Tai F.-I.; Sterner O.; Andersson O.; Ekblad T.; Ederth T. pH-control of the protein resistance of thin hydrogel gradient films. Soft Matter 2014, 10 (32), 5955–5964. 10.1039/C4SM00833B. 24987939

[ref29] Ederth T.; Ekblad T. Swelling of Thin Poly(ethylene glycol)-Containing Hydrogel Films in Water Vapor—A Neutron Reflectivity Study. Langmuir 2018, 34 (19), 5517–5526. 10.1021/acs.langmuir.8b00177. 29672068

[ref30] Larsson A.; Ekblad T.; Andersson O.; Liedberg B. Photografted Poly(ethylene glycol) Matrix for Affinity Interaction Studies. Biomacromolecules 2007, 8 (1), 287–295. 10.1021/bm060685g. 17206819

[ref31] Ekblad T.; Bergström G.; Ederth T.; Conlan S. L.; Mutton R.; Clare A. S.; Wang S.; Liu Y.; Zhao Q.; D’Souza F.; Donnelly G. T.; Willemsen P. R.; Pettitt M. E.; Callow M. E.; Callow J. A.; Liedberg B. Poly(ethylene glycol)-Containing Hydrogel Surfaces for Antifouling Applications in Marine and Freshwater Environments. Biomacromolecules 2008, 9 (10), 2775–2783. 10.1021/bm800547m. 18759475

[ref32] Nagy B.; Campana M.; Khaydukov Y. N.; Ederth T. Structure and pH-Induced Swelling of Polymer Films Prepared from Sequentially Grafted Polyelectrolytes. Langmuir 2022, 38 (5), 1725–1737. 10.1021/acs.langmuir.1c02784. 35081310 PMC8830213

[ref33] Yandi W.; Nagy B.; Skallberg A.; Uvdal K.; Zimmermann R.; Liedberg B.; Ederth T. Polyampholytic Poly(AEMA-co-SPMA) Thin Films and Their Potential for Antifouling Applications. ACS Appl. Polym. Mater. 2021, 3 (11), 5361–5372. 10.1021/acsapm.1c00383.

[ref34] Ekblad T.; Andersson O.; Tai F.-I.; Ederth T.; Liedberg B. Lateral Control of Protein Adsorption on Charged Polymer Gradients. Langmuir 2009, 25 (6), 3755–3762. 10.1021/la803443d. 19708252

[ref35] Tai F.-I.; Sterner O.; Andersson O.; Ekblad T.; Ederth T. Interaction Forces on Polyampholytic Hydrogel Gradient Surfaces. ACS Omega 2019, 4 (3), 5670–5681. 10.1021/acsomega.9b00339. 31459721 PMC6648739

[ref36] de Gennes P. G. Conformations of Polymers Attached to an Interface. Macromolecules 1980, 13 (5), 1069–1075. 10.1021/ma60077a009.

[ref37] Milner S. T.; Witten T. A.; Cates M. E. Theory of the grafted polymer brush. Macromolecules 1988, 21 (8), 2610–2619. 10.1021/ma00186a051.

[ref38] Zhulina E. B.; Borisov O. V.; Priamitsyn V. A. Theory of steric stabilization of colloid dispersions by grafted polymers. J. Colloid Interface Sci. 1990, 137 (2), 495–511. 10.1016/0021-9797(90)90423-L.

[ref39] Mavrantzas V. G. Using Monte Carlo to Simulate Complex Polymer Systems: Recent Progress and Outlook. Frontiers in Physics 2021, 9, 661367 10.3389/fphy.2021.661367.

[ref40] Dimitrov D. I.; Milchev A.; Binder K. Polymer brushes in solvents of variable quality: Molecular dynamics simulations using explicit solvent. J. Chem. Phys. 2007, 127 (8), 084905 10.1063/1.2768525. 17764292

[ref41] Kent M. S.; Lee L. T.; Factor B. J.; Rondelez F.; Smith G. S. Tethered chains in good solvent conditions: An experimental study involving Langmuir diblock copolymer monolayers. J. Chem. Phys. 1995, 103 (6), 2320–2342. 10.1063/1.469707.

[ref42] Kent M. S.; Majewski J.; Smith G. S.; Lee L. T.; Satija S. Tethered chains in theta solvent conditions: An experimental study involving Langmuir diblock copolymer monolayers. J. Chem. Phys. 1998, 108 (13), 5635–5645. 10.1063/1.475952.

[ref43] Kent M. S.; Majewski J.; Smith G. S.; Lee L. T.; Satija S. Tethered chains in poor solvent conditions: An experimental study involving Langmuir diblock copolymer monolayers. J. Chem. Phys. 1999, 110 (7), 3553–3565. 10.1063/1.478223.

[ref44] Rånby B. G.; Rabek J. F. Photodegradation, Photo-oxidation, and Photostabilization of Polymers; Wiley: New York, 1975.

[ref45] Yousif E.; Haddad R. Photodegradation and photostabilization of polymers, especially polystyrene: review. SpringerPlus 2013, 2 (1), 398 10.1186/2193-1801-2-398. 25674392 PMC4320144

[ref46] Li L.; Jakowski J.; Do C.; Hong K. Deuteration and Polymers: Rich History with Great Potential. Macromolecules 2021, 54 (8), 3555–3584. 10.1021/acs.macromol.0c02284.

[ref47] Majkrzak C. F.; Berk N. F. Exact determination of the phase in neutron reflectometry. Phys. Rev. B 1995, 52 (15), 10827–10830. 10.1103/PhysRevB.52.10827. 9980180

[ref48] Larsson A.; Du C.-X.; Liedberg B. UV-Patterned Poly(ethylene glycol) Matrix for Microarray Applications. Biomacromolecules 2007, 8 (11), 3511–3518. 10.1021/bm700707s. 17910496

[ref49] Faxälv L.; Ekblad T.; Liedberg B.; Lindahl T. L. Blood compatibility of photografted hydrogel coatings. Acta Biomaterialia 2010, 6 (7), 2599–2608. 10.1016/j.actbio.2009.12.046. 20045090

[ref50] Cubitt R.; Fragneto G. D17: the new reflectometer at the ILL. Appl. Phys. A: Mater. Sci. Process. 2002, 74 (1), s329–s331. 10.1007/s003390201611.

[ref51] Durant J. H.; Wilkins L.; Cooper J. F. K. Optimizing experimental design in neutron reflectometry. J. Appl. Crystallogr. 2022, 55 (4), 769–781. 10.1107/S1600576722003831. 35974737 PMC9348865

[ref52] Bjorck M.; Andersson G. GenX: an extensible X-ray reflectivity refinement program utilizing differential evolution. J. Appl. Crystallogr. 2007, 40 (6), 1174–1178. 10.1107/S0021889807045086.

[ref53] Campbell R. A. Recent advances in resolving kinetic and dynamic processes at the air/water interface using specular neutron reflectometry. Curr. Opin. Colloid Interface Sci. 2018, 37, 49–60. 10.1016/j.cocis.2018.06.002.

[ref54] Shull K. R. Theory of end-adsorbed polymer brushes in polymeric matrices. J. Chem. Phys. 1991, 94 (8), 5723–5738. 10.1063/1.460456.

[ref55] Zhulina E. B.; Borisov O. V.; Birshtein T. M. Structure of grafted polyelectrolyte layer. J. Phys. II France 1992, 2 (1), 63–74. 10.1051/jp2:1992113.

[ref56] Murdoch T. J.; Humphreys B. A.; Willott J. D.; Gregory K. P.; Prescott S. W.; Nelson A.; Wanless E. J.; Webber G. B. Specific Anion Effects on the Internal Structure of a Poly(N-isopropylacrylamide) Brush. Macromolecules 2016, 49 (16), 6050–6060. 10.1021/acs.macromol.6b01001.

[ref57] Yunogami T.; Mizutani T.; Suzuki K.; Nishimatsu S. Radiation Damage in SiO_2_/Si Induced by VUV Photons. Jpn. J. Appl. Phys. 1989, 28, 2172–2176. 10.1143/JJAP.28.2172.

[ref58] Esebamen O. X. Effect of UV radiation surface damage on silicon position sensitive photodetector. Optik 2016, 127 (2), 599–602. 10.1016/j.ijleo.2015.09.074.

[ref59] Zhulina E. B.; Borisov O. V.; Pryamitsyn V. A.; Birshtein T. M. Coil-globule type transitions in polymers. 1. Collapse of layers of grafted polymer chains. Macromolecules 1991, 24 (1), 140–149. 10.1021/ma00001a023.

[ref60] Shim D. F. K.; Cates M. E. Finite extensibility and density saturation effects in the polymer brush. J. Phys. (Paris) 1989, 50 (24), 3535–3551. 10.1051/jphys:0198900500240353500.

[ref61] Baranowski R.; Whitmore M. D. Numerical self-consistent field study of tethered chains in Θ solvent. J. Chem. Phys. 1998, 108 (23), 9885–9892. 10.1063/1.476427.

[ref62] Cao N.; Zhao Y.; Chen H.; Huang J.; Yu M.; Bao Y.; Wang D.; Cui S. Poly(ethylene glycol) Becomes a Supra-Polyelectrolyte by Capturing Hydronium Ions in Water. Macromolecules 2022, 55 (11), 4656–4664. 10.1021/acs.macromol.2c00014.

[ref63] Wang R. L. C.; Kreuzer H. J.; Grunze M. The interaction of oligo(ethylene oxide) with water: a quantum mechanical study. Phys. Chem. Chem. Phys. 2000, 2 (16), 3613–3622. 10.1039/b002593n.

[ref64] Di Fonzo S.; Bellich B.; Gamini A.; Quadri N.; Cesàro A. PEG hydration and conformation in aqueous solution: Hints to macromolecular crowding. Polymer 2019, 175, 57–64. 10.1016/j.polymer.2019.05.004.

[ref65] Schneck E.; Berts I.; Halperin A.; Daillant J.; Fragneto G. Neutron reflectometry from poly (ethylene-glycol) brushes binding anti-PEG antibodies: Evidence of ternary adsorption. Biomaterials 2015, 46, 95–104. 10.1016/j.biomaterials.2014.12.041. 25678119

[ref66] Schneck E.; Schollier A.; Halperin A.; Moulin M.; Haertlein M.; Sferrazza M.; Fragneto G. Neutron Reflectometry Elucidates Density Profiles of Deuterated Proteins Adsorbed onto Surfaces Displaying Poly(ethylene glycol) Brushes: Evidence for Primary Adsorption. Langmuir 2013, 29 (46), 14178–14187. 10.1021/la403355r. 24144259

[ref67] Mohammadi E.; Joshi S. Y.; Deshmukh S. A. A review of computational studies of bottlebrush polymers. Comput. Mater. Sci. 2021, 199, 110720 10.1016/j.commatsci.2021.110720.

[ref68] Li Z.; Tang M.; Liang S.; Zhang M.; Biesold G. M.; He Y.; Hao S.-M.; Choi W.; Liu Y.; Peng J.; Lin Z. Bottlebrush polymers: From controlled synthesis, self-assembly, properties to applications. Prog. Polym. Sci. 2021, 116, 101387 10.1016/j.progpolymsci.2021.101387.

[ref69] Varga I.; Mészáros R.; Makuška R.; Claesson P. M.; Gilányi T. Effect of Graft Density on the Nonionic Bottle Brush Polymer/Surfactant Interaction. Langmuir 2009, 25 (19), 11383–11389. 10.1021/la901499x. 19736986

[ref70] Liu X.; Dedinaite A.; Nylander T.; Dabkowska A. P.; Skoda M.; Makuska R.; Claesson P. M. Association of anionic surfactant and physisorbed branched brush layers probed by neutron and optical reflectometry. J. Colloid Interface Sci. 2015, 440, 245–252. 10.1016/j.jcis.2014.11.002. 25460712

[ref71] Jungmann P.; Kreer T.; Sommer J.-U.; Paturej J. Conformational Properties of End-Grafted Bottlebrush Polymers. Macromolecules 2021, 54 (1), 161–169. 10.1021/acs.macromol.0c01586.

[ref72] Yandi W.; Mieszkin S.; Martin-Tanchereau P.; Callow M. E.; Callow J. A.; Tyson L.; Liedberg B.; Ederth T. Hydration and Chain Entanglement Determines the Optimum Thickness of Poly(HEMA-co-PEG10MA) Brushes for Effective Resistance to Settlement and Adhesion of Marine Fouling Organisms. ACS Appl. Mater. Interfaces 2014, 6 (14), 11448–11458. 10.1021/am502084x. 24945705

[ref73] Henze M.; Mädge D.; Prucker O.; Rühe J. “Grafting Through”: Mechanistic Aspects of Radical Polymerization Reactions with Surface-Attached Monomers. Macromolecules 2014, 47 (9), 2929–2937. 10.1021/ma402607d.

[ref74] Menzies D. J.; Nelson A.; Shen H.-H.; McLean K. M.; Forsythe J. S.; Gengenbach T.; Fong C.; Muir B. W. An X-ray and neutron reflectometry study of ‘PEG-like’ plasma polymer films. Journal of The Royal Society Interface 2012, 9 (70), 1008–1019. 10.1098/rsif.2011.0509. 21957120 PMC3306642

[ref75] Akers P. W.; Nelson A. R. J.; Williams D. E.; McGillivray D. J. Formation of hydrated layers in PMMA thin films in aqueous solution. Appl. Surf. Sci. 2015, 353, 829–834. 10.1016/j.apsusc.2015.06.199.

[ref76] Hu N.; Chen C.; Metwalli E.; Bießmann L.; Herold C.; Fu J.; Cubitt R.; Zhong Q.; Müller-Buschbaum P. Hydration and Thermal Response Kinetics of a Cross-Linked Thermoresponsive Copolymer Film on a Hydrophobic PAN Substrate Coating Probed by In Situ Neutron Reflectivity. Langmuir 2021, 37 (22), 6819–6829. 10.1021/acs.langmuir.1c00931. 34043364

