# Supplementary material for: Structure of Self-Initiated Photopolymerized Films: A Comparison of Models
Source: Langmuir. 2022 Nov 3;38(45):14004–15. doi: 10.1021/acs.langmuir.2c02396 (PMC9671054; doi:10.1021/acs.langmuir.2c02396)
Supplement: Supplementary file 1 — la2c02396_si_001.pdf [file la2c02396_si_001.pdf]

Supporting Information

Structure of self-initiated photopolymerized films: A comparison of models

Béla Nagy[1], Tobias Ekblad[1,†], Giovanna Fragneto[2], Thomas Ederth[1,*]

[1] *Division of Biophysics and Bioengineering, Department of Physics, Chemistry and Biology, Linköping University, SE-581 83 Linköping, Sweden*

[2] *Institut Laue-Langevin, 71 avenue des Martyrs, BP 156, 38042 Grenoble, France*

* Email: thomas.ederth@liu.se

† Present address: HemoCue AB, Ängelholm, Sweden

Supporting Information Table of Contents *Page*

Acquisition details for the reflectometry experiments S2
Effects of UV illumination on the native oxide layer on Si S2
Constraining the amount of polymer S3
Figure of merit values S5
Fit Comparison, Sample Si1 S6
Fit Comparison, Sample Si2 S9
Fit Comparison, Sample Au1 S12
Deuterated layer parameters S15
Grafting density and the swelling of the layers S16
Comparing polymer volume fraction profiles of different models S16
Infrared spectroscopy for monitoring hydration S17
Appendix 1 Python script S18
Appendix 2 Python script S18

Acquisition details for the reflectometry experiments

Table S1. Measurement times for the different angles and contrasts. 1st refers to measurement after grafting of the p(HEMA-*co*-PEG$_{10}$MA) layer, 2nd refers to measurement after grafting of the dMAA layer.

Contrast	SLD	Angle	Si1 1st	Si1 2nd	Si2 1st	Si2 2nd	Au1 1st	Au2 2nd
	(10^{-6} Å^{-2})	(°)			(s)			
D_2O	6.38	0.7	2700 s	1800 s	2700 s	1200 s	900 s	900 s
		3.0	3600 s	1800 s	5400 s	1800 s	1800 s	1800 s
CMAu	4.5	0.7	n.a.	n.a.	n.a.	n.a.	900 s	900 s
		3.0	n.a.	n.a.	n.a.	n.a.	1800 s	1800 s
CM4	4.0	0.7	1800 s	2400 s	1800 s	2400 s	n.a.	n.a.
		3.0	3600 s	2400 s	3600 s	2400 s	n.a.	n.a.
CMSi	2.07	0.7	7200 s	7200 s	7200 s	7200 s	900 s	900 s
		3.0	7200 s	7200 s	5400 s	7200 s	1800 s	1800 s
H_2O	-0.57	0.7	3600 s	3600 s	n.a.	n.a.	n.a.	n.a.
		3.0	3600 s	3600 s	n.a.	n.a.	n.a.	n.a.
Dry	0	0.7	3600 s	3600 s	3600 s	3600 s	1800 s	900 s
		3.0	3600 s	3600 s	3600 s	3600 s	1800 s	900 s

Effects of UV illumination on the native oxide layer on Si

Using spectroscopic ellipsometry, we have tested the effect of the used UV-radiation on the thickness of the native oxide layer on silicon wafers. In these tests, pure water was replacing the aqueous monomer solutions, but otherwise the tests were carried out under conditions identical to those used during polymerization. The measurements were conducted using a JA Woollam iSE ellipsometer on a 25×15 mm^2 silicon wafer. The wafer was cleaned by the TL-1 procedure (aka RCA SC-1, sample immersed for 5 min in a 5:1:1 mixture of H_2O, 25% NH_3 and 30% H_2O_2 at 85°C). The sample then was irradiated for 4 minutes twice according to the procedure described in the paper using pure water instead of a monomer solution. The ellipsometric angles (Ψ and Δ) were recorded before each irradiation step and after the last irradiation, for 190 wavelengths from 400 nm to 1000 nm at a 70° angle of incidence. The data was then fit with a model consisting of a Si substrate and a native oxide layer using tabulated data supplied with the ellipsometer for the refractive indices. The resulting native oxide layer thicknesses are presented in Table S 2.

Table S2. Native oxide thicknesses measured with spectroscopic ellipsometry after UV irradiation of a Si wafer.

**Irradiation time (min)**	0	4	8
**Thickness (Å)**	8.8 ± 0.3	9.4 ± 0.3	10.4 ± 0.2

Constraining the amount of polymer

The polymer amount in the dry sample can be written as $M = \rho_{poly} h_{dry}$, where ρ_{poly} is the scattering length density of the polymer and h_{dry} is the thickness of the dry layer. The polymer amount in the wet sample is $M = \rho_{poly} \int_0^{\infty} \phi(z)dz$, where ρ_{poly} is the SLD of the polymer and $\phi(z)$ is the polymer volume fraction profile of the brush. We have modelled our data with three profiles: the stretched parabolic profile of a brush in good solvent, a slab profile with sigmoidal roughness and a Gaussian model analogous to a stretched charged polyelectrolyte brush. The parabolic model has three parameters: ϕ_0 the polymer volume fraction at the interface, h_p the height of the brush and α a parameter describing the shape of the profile. The sigmoidal model has three parameters: ϕ_0 the polymer volume fraction at the interface, h_s the thickness of the slab and σ_s the roughness parameter. While the Gaussian profile has two parameters ϕ_0 the polymer volume fraction at the interface and σ_g the characteristic length. The formulas describing the profiles, the integrals of the profiles and the first moment of the profiles are shown in Table S 3.

Table S3. The formulas, the integrals and the first moments of the polymer volume fraction profiles of the different models used to describe the neutron reflectivity data.

MODEL	$\boldsymbol{\phi(z)}$	$\int_0^{\infty} \boldsymbol{\phi(z)dz}$	$\int_0^{\infty} \boldsymbol{z\,\phi(z)dz}$
**PARABOLIC MODEL**	$\phi_0\left(1-\left(\frac{z}{h_p}\right)^2\right)^{\alpha}$	$\frac{\phi_0 h_p \sqrt{\pi}\,\Gamma(1+\alpha)}{2\,\Gamma\left(\frac{3}{2}+\alpha\right)}$	$\frac{\phi_0 h_p^2}{2(\alpha+1)}$
**SIGMOIDAL MODEL**	$\frac{\phi_0}{2}\left(\mathrm{erf}\left(\frac{h_s-z}{\sigma_s}\right)+1\right)$	$\frac{\phi_0}{2}\left(h_s\left(\mathrm{erf}\left(\frac{h_s}{\sigma_s}\right)+1\right)+\frac{\sigma_s e^{-\left(\frac{h_s}{\sigma_s}\right)^2}}{\sqrt{\pi}}\right)$	$\frac{\phi_0}{8}\left((\sigma_s^2+2h_s^2)\left(1+erf\left(\frac{h_s}{\sigma_s}\right)\right)+\frac{2h_s\sigma_s}{\sqrt{\pi}}e^{-\left(\frac{h_s}{\sigma_s}\right)^2}\right)$
**GAUSSIAN MODEL**	$\phi_0 e^{-\left(\frac{z}{\sigma_g}\right)^2}$	$\frac{\phi_0\sqrt{\pi}\sigma_g}{2}$	$\frac{\phi_0\sigma_g^2}{2}$

In case of the Gaussian model the parameter $\sigma_g = 2h_{dry}/\sqrt{\pi}\phi_0$ can be easily calculated and implemented into the fitting routine. Implementing the constraints into the other two models require more elaborate considerations. In case of the stretched parabolic profile determining h_p is straightforward, however, we opted to keep h_p as a fitting parameter and constrain α, as the total layer thickness can be determined from the period of the Kiessig fringes. To determine the value of α one needs to solve:

$$F(\alpha) = h_{dry}/\phi_0 h_p = \sqrt{\pi}\,\Gamma(1+\alpha)/2\,\Gamma\left(\frac{3}{2}+\alpha\right) \quad \text{(S1)}$$

The plot of $F(\alpha)$ is presented in Figure S 1. Inverting this function requires numerical approximations. Since it is a monotonously decreasing function for $\alpha > 0$ and $\lim_{\alpha\to\infty} F(\alpha) \to 0$, one can solve the $F'(\alpha) = \sqrt{\pi}\,\Gamma(1+\alpha)/2\,\Gamma\left(\frac{3}{2}+\alpha\right) - h_{dry}/\phi_0 h_p = 0$ equation with the use of the bisection method. The python code for this procedure is presented in Appendix 1.

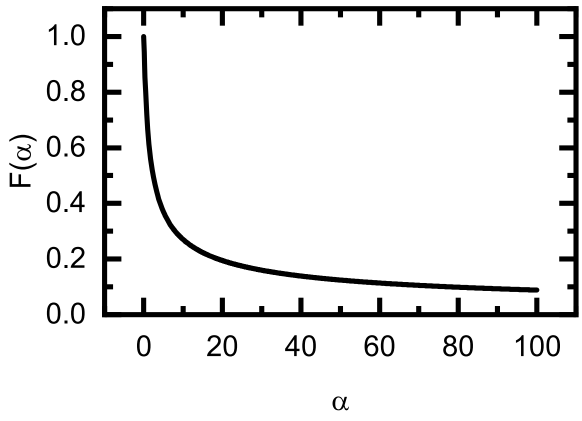

Figure S1. Plot of the $\boldsymbol{F(\alpha)}$ function.

The sigmoidal model represents the default roughness model used in reflectometry, however, here the roughness parameter represents the shape of the layer. When $h_s < 3\sigma_s$ one must consider the shape of the profile when integrating the area under the curve and use the formula displayed in Table S 3, instead of treating the problem as a slab profile. The differences in the profile and the integration are illustrated in Figure S 2. Since the integral function is also monotonous for values $h > 0$ we used a similar procedure as with the stretched exponential profile. In case of $h_s > 3\sigma_s$ the value of σ_s is not determined by the formula presented in Table S 3, this requires that the value of h_s is calculated using the constraint. The python code for this procedure is presented in Appendix 2.

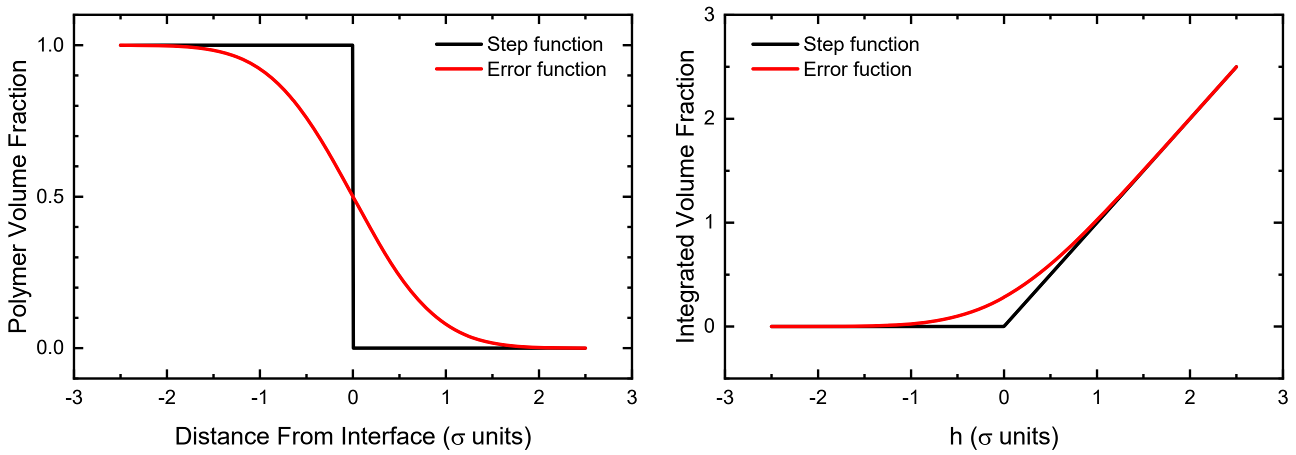

Figure S2. Shape of the sigmoidal roughness (red) compared to a step profile(black) (left). The value of the integrated volume fraction for the two profiles as a function of the layer thickness.

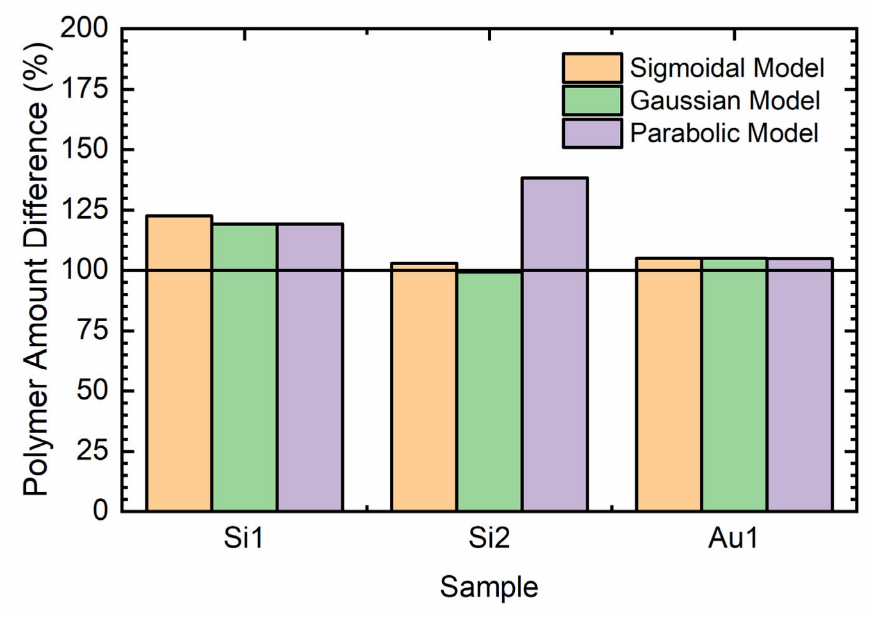

Figure S3. Ratio of the dry and hydrated amounts of polymer calculated by numerically integrating the volume fraction profiles. 100% corresponds to the dry polymer amount.

Figure of merit values

Table S4. Figure of merit values for the different models for each sample, after fitting the models to the hydrogenated p(HEMA-*co*-PEG$_{10}$MA) layer. Note that the hydrogenated layer by itself was not measured on sample Au2.

**Sample**	**Figure of merit (a.u.)**		
	Parabolic	Sigmoidal	Gaussian
**Si1**	11.79	11.60	11.91
**Si2**	11.15	11.22	11.33
**Au1**	3.23	3.66	3.16

Fit Comparison

Sample Si1

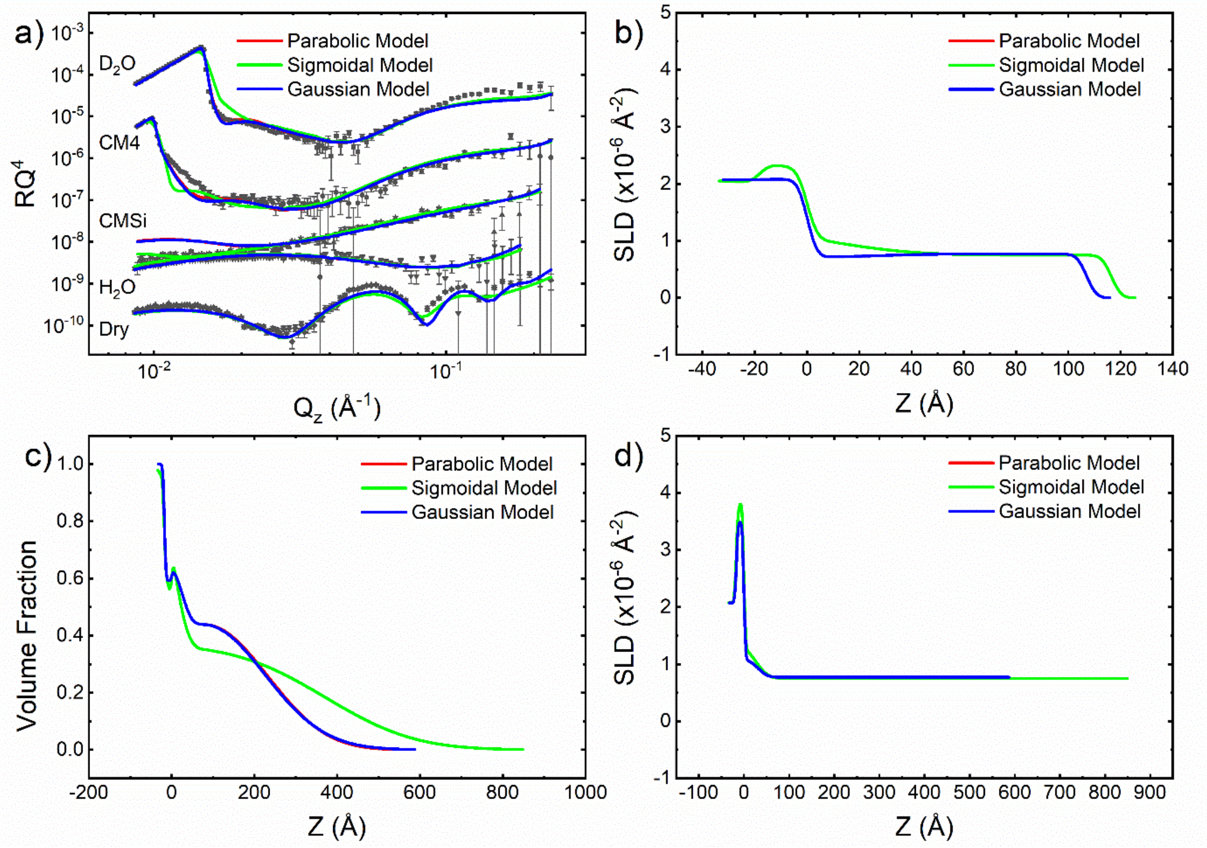

Figure S4. Comparison of the different models for the hydrogenated layer on sample Si1. The measured data (dots) is presented in a) with the resulting curves from the parabolic brush (red), sigmoidal (blue) and Gaussian (green) models. The scattering length density profiles, measured on the dry sample, are shown in b). For the hydrated polymer, c) is the volume fraction and d) the scattering length density of the non-aqueous components. In b)-d), the red curves are largely hidden behind the blue.

Table S5. Fit parameters for the different models describing the hydrogenated layer on sample Si1. The displayed errors are estimates calculated from a ± 5% increase in the FOM. In the table, $\boldsymbol{d}$ is layer thickness, $\boldsymbol{\sigma}$ is interfacial roughness, $\boldsymbol{\rho}$ is SLD, $\boldsymbol{\phi}$ volume fraction, $\boldsymbol{\phi_0}$ volume fraction at the solid surface, and $\boldsymbol{h}$ is the brush height, $\boldsymbol{\alpha}$ is the stretching exponent for parabolic profile, $\boldsymbol{\sigma_s}$ is the roughness of the sigmoidal layer, and $\boldsymbol{\sigma_g}$ is the characteristic length of the Gaussian profile.

Parameter	Parabolic model	Sigmoidal model	Gaussian model
$\boldsymbol{d_{SiO2}}$ (Å)	18±2	18 ± 2	18 ± 2
$\boldsymbol{\sigma_{SiO2}}$ (Å)	3.0 ± 1.9	3 ± 2	3.0 ± 1.9
$\boldsymbol{\phi_{SiO2}}$	0.60 ± 0.06	0.69 ± 0.06	0.60 ± 0.06
$\boldsymbol{d_{Silane}}$ (Å)	27 ± 5	10 ± 4	27 ± 5
$\boldsymbol{\sigma_{Silane}}$ (Å)	17 ± 9	25 ± 5	17 ± 8
$\boldsymbol{\phi_{Silane}}$	0.64 ± 0.05	0.86 ± 0.11	0.64 ± 0.05
$\boldsymbol{\rho_{Silane}}(\times \mathbf{10^{-6}}\ \text{Å}^{-2})$	1.1 ± 0.2	1.4 ± 0.2	1.1 ± 0.2
$\boldsymbol{\rho_{Poly}}\ (\times \mathbf{10^{-6}}\ \text{Å}^{-2})$	0.77 ± 0.12	0.75 ± 0.10	0.77 ± 0.11
$\boldsymbol{\phi_0}$	0.44 ± 0.04	0.37 ± 0.05	0.44 ± 0.04
$\boldsymbol{\alpha}$	13 ± 11	n.a.	n.a.
$\boldsymbol{h_p}$ (Å)	800 ± 300	n.a.	n.a.
$\boldsymbol{\sigma_s}$ (Å)	n.a.	240 ± 170	n.a.
$\boldsymbol{h_s}$ (Å)	n.a.	280 ± 90	n.a.
$\boldsymbol{\sigma_g}$ (Å)	n.a.	n.a.	210 ± 40
$\boldsymbol{h_g}$ (Å)	n.a.	n.a.	620 ± 110
$\boldsymbol{d_{dry}}$ (Å)	80 ± 6	106 ± 8	80 ± 7
$\boldsymbol{\sigma_{dry}}$ (Å)	3 ± 19	3 ± 18	3 ± 20
$\boldsymbol{d_{wet}}$ (Å)	228	382	233
**Swelling**	285%	359%	291%
**HEMA Content**	20 ± 40%	10 ± 40%	20 ± 40%

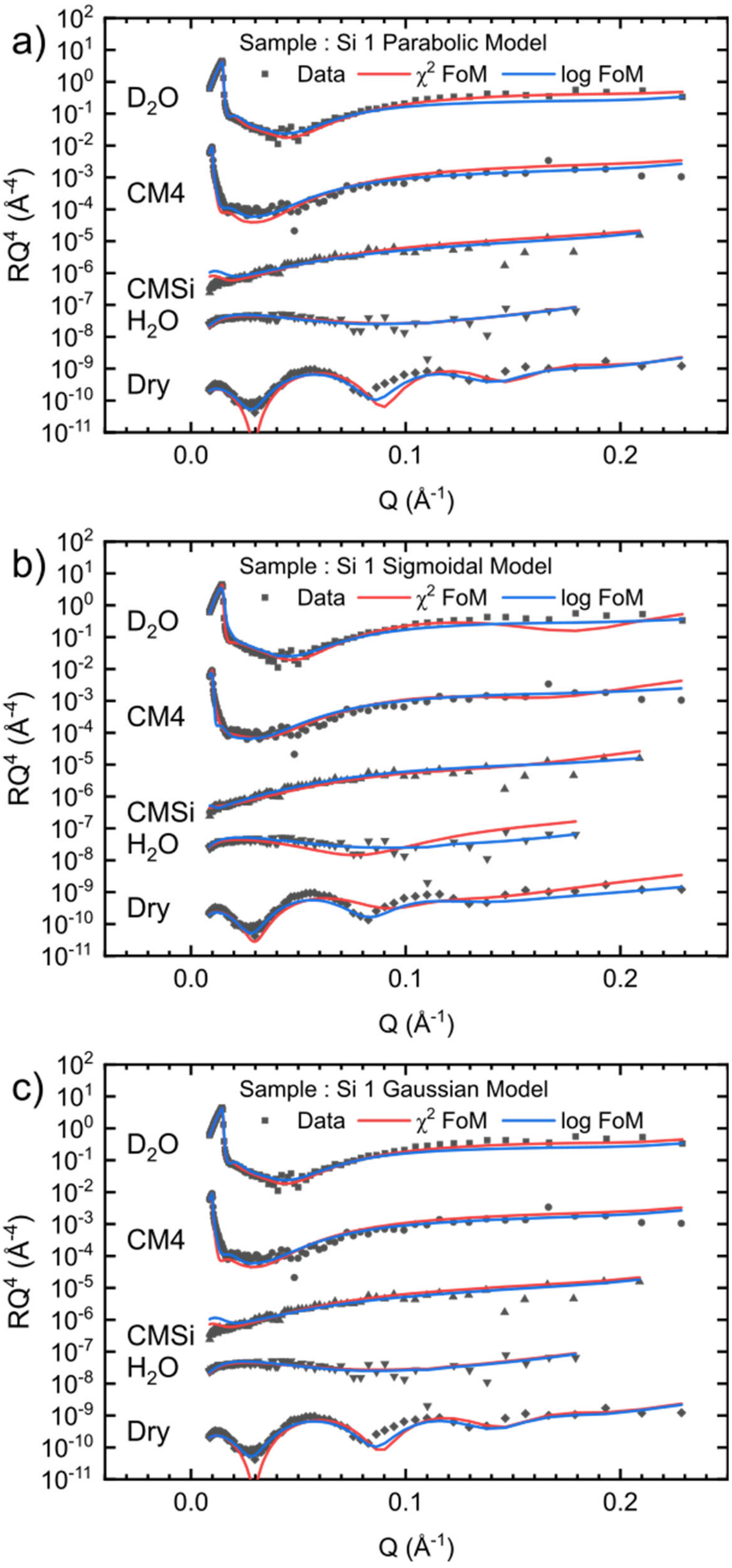

Figure S5. Comparison of the modelling using the chi-square figure of merit (red) and the logarithmic figure of merit (blue) for samples Si 1.

Sample Si2

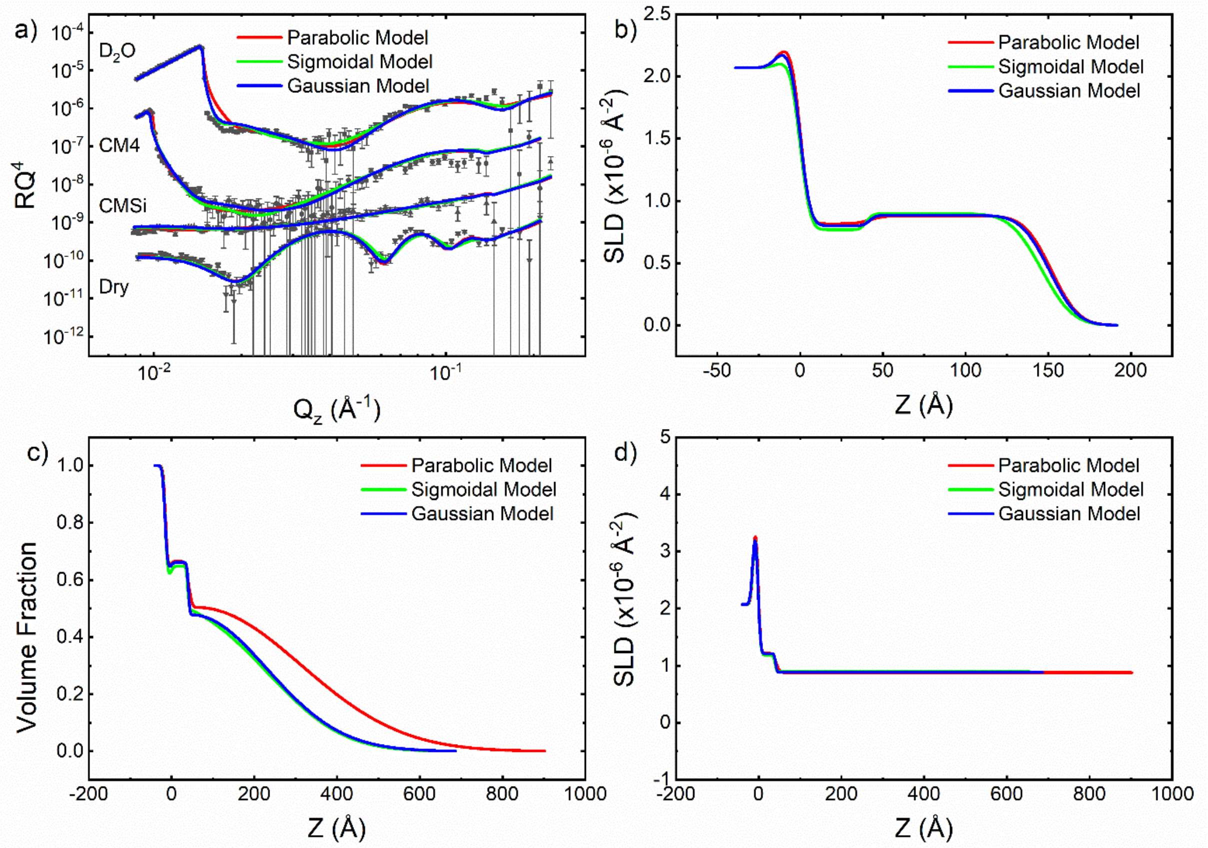

Figure S6. Comparison of the different models for the hydrogenated layer on sample Si2. The measured data (dots) is presented in a) with the resulting curves from the parabolic brush (red), sigmoidal (blue) and Gaussian (green) models. The scattering length density profiles, measured on the dry sample, are shown in b). For the hydrated polymer, c) is the volume fraction and d) the scattering length density of the non-aqueous components.

Table S6. Fit parameters for the different models describing the hydrogenated layer on sample Si2. The displayed errors are estimates calculated from a ± 5% increase in the FOM. See caption of Table S 5 for explanation of the parameters in the table.

**Parameter**	**Parabolic model**	**Sigmoidal model**	**Gaussian model**
d_{SiO2} (Å)	14.4 ± 1.3	14.4 ± 1.4	14.6 ± 1.4
σ_{SiO2} (Å)	4 ± 2	5 ± 2	5 ± 2
ϕ_{SiO2}	0.64 ± 0.06	0.61 ± 0.07	0.64 ± 0.07
d_{Silane} (Å)	42 ± 4	40 ± 4	42 ± 5
σ_{Silane} (Å)	7 ± 12	3 ± 5	3 ± 4
ϕ_{Silane}	0.67 ± 0.02	0.65 ± 0.02	0.66 ± 0.02
$\rho_{Silane}(\times 10^{-6}$ Å$^{-2})$	1.22 ± 0.09	1.19 ± 0.09	1.21 ± 0.09
ρ_{Poly} $(\times 10^{-6}$ Å$^{-2})$	0.88 ± 0.07	0.90 ± 0.07	0.88 ± 0.07
ϕ_0	0.50 ± 0.3	0.55 ± 0.04	0.48 ± 0.02
α	60 ± 20	n.a.	n.a.
h_p (Å)	2000 ± 200	n.a.	n.a.
σ_s (Å)	n.a.	206 ± 15	n.a.
h_s (Å)	n.a.	190 ± 30	n.a.
σ_g (Å)	n.a.	n.a.	260 ± 20
h_g (Å)	n.a.	n.a.	770 ± 70
d_{dry} (Å)	110 ± 6	106 ± 6	13 ± 10
σ_{dry} (Å)	13 ± 10	15 ± 11	109 ± 6
d_{wet} (Å)	278.76	284.56	289.63
**Swelling**	252%	269%	266%
**HEMA Content**	60 ± 20%	70 ± 20%	60 ± 30%

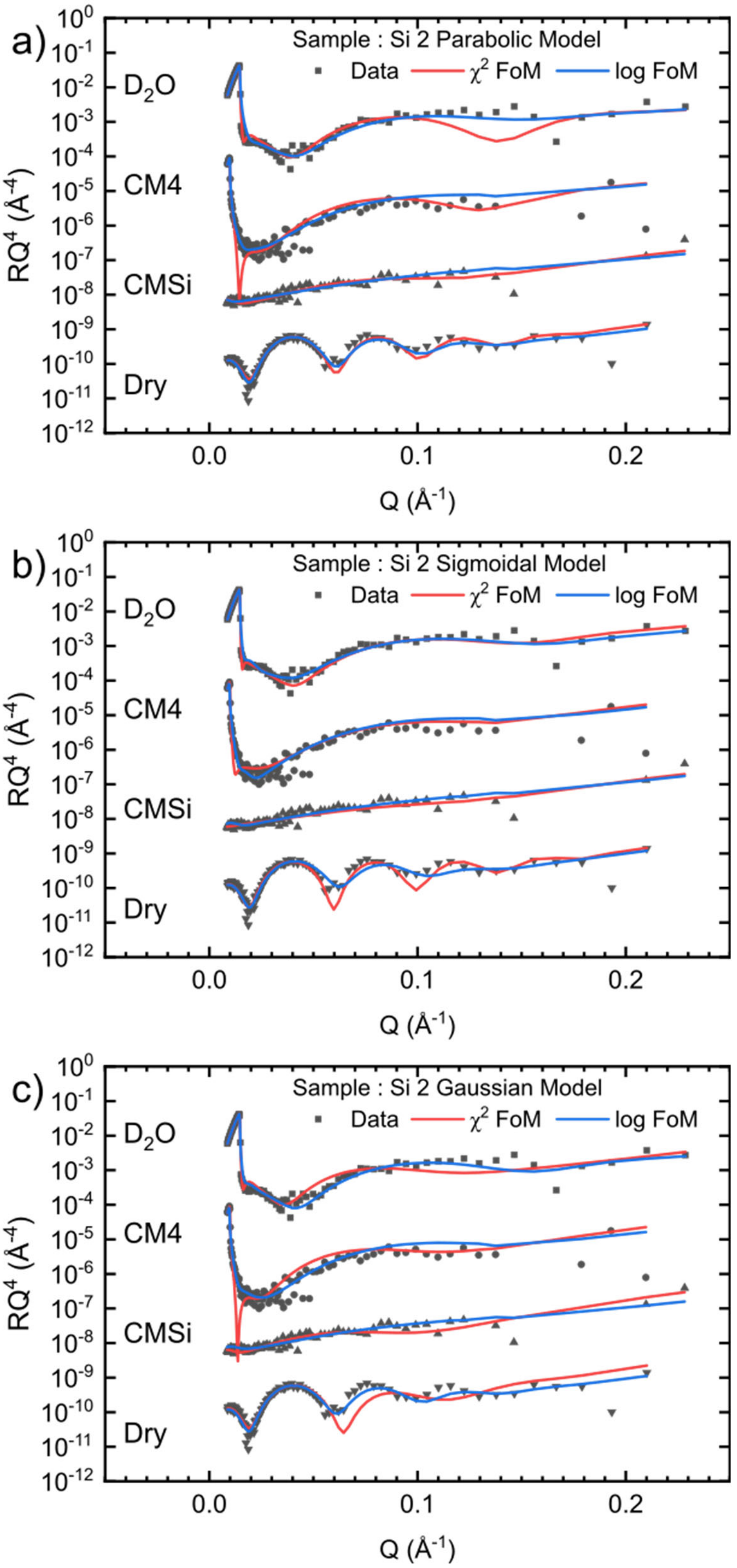

Figure S7. Comparison of the modelling using the chi-square figure of merit (red) and the logarithmic figure of merit (blue) for samples Si 2.

Sample Au 1

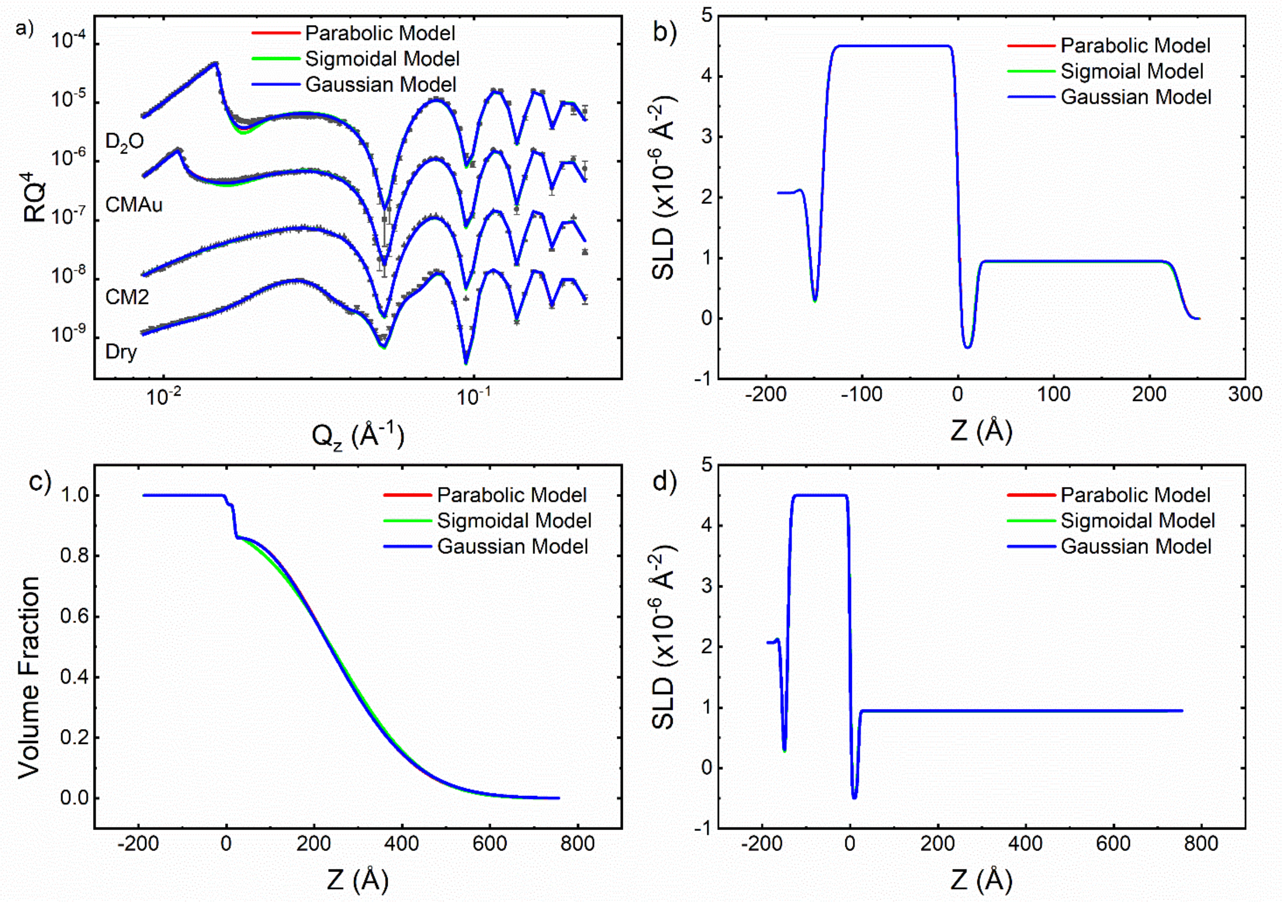

Figure S8. Comparison of the different models for the hydrogenated layer on sample Au1. The measured data (dots) is presented in a) with the resulting curves from the parabolic brush (red), sigmoidal (blue) and Gaussian (green) models. The scattering length density profiles, measured on the dry sample, are shown in b). For the hydrated polymer, c) is the volume fraction and d) the scattering length density of the non-aqueous components.

Table S7. Fit parameters for the different models describing the hydrogenated layer on sample Au1. The displayed errors are estimates calculated from a ± 5% increase in the FOM. See caption of Table S 5 for explanation of the parameters in the table.

Parameter	Parabolic model	Sigmoidal model	Gaussian model
d_{SiO2} (Å)	5.0 ± 1.8	5 ± 2	5.0 ± 1.8
σ_{SiO2} (Å)	5.9 ± 0.9	5.9 ± 0.9	5.9 ± 0.8
d_{Ti} (Å)	10.1 ± 0.6	10.2 ± 0.7	10.1 ± 0.6
d_{Au} (Å)	143.1 ± 0.7	143.1 ± 0.7	143.1 ± 0.7
d_{Thiol} (Å)	18.7 ± 1.3	18.1 ± 1.5	18.7± 1.3
σ_{Thiol} (Å)	3.0 ± 0.8	3.0 ± 0.8	3.0 ± 0.8
ϕ_{Thiol}	0.97 ± 0.03	0.97 ± 0.03	-0.50 ± 013
ρ_{Thiol} ($\times 10^{-6}$ Å^{-2})	-0.50 ± 0.13	-0.50 ± 0.14	0.97 ± 0.03
ρ_{Poly} ($\times 10^{-6}$ Å^{-2})	0.95 ± 0.09	0.94 ± 0.10	0.95 ± 0.09
ϕ_0	0.86 ± 0.03	0.93 ± 0.05	0.86 ± 0.03
α	30 ± 20	n.a.	n.a.
h_p (Å)	1600 ± 900	n.a.	n.a.
σ_s (Å)	n.a.	220 ± 30	n.a.
h_s (Å)	n.a.	220 ± 30	n.a.
σ_g (Å)	n.a.	n.a.	280 ± 19
h_g (Å)	n.a.	n.a.	840 ± 60
d_{dry} (Å)	213 ± 7	213 ± 11	213 ± 7
σ_{dry} (Å)	6 ± 11	7 ± 12	6 ± 11
d_{wet} (Å)	313	318	315
**Swelling**	147%	149%	148%
**HEMA Content**	80 ± 30%	80 ± 40%	80 ± 30%

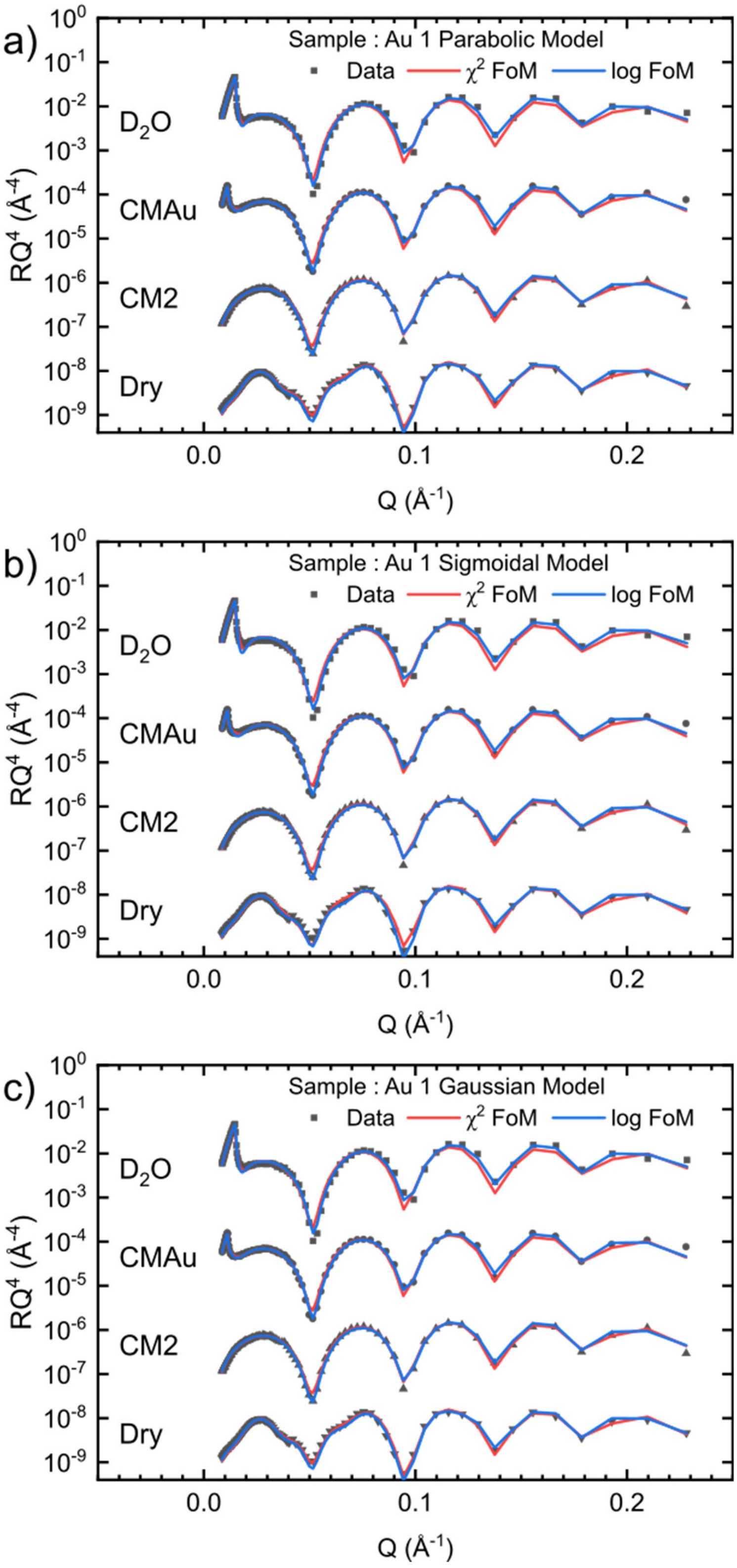

Figure S9. Comparison of the modelling using the chi-square figure of merit (red) and the logarithmic figure of merit (blue) for sample Au 1.

Deuterated layer parameters

Table S8. Fit parameters for the different models describing the deuterated layers. The displayed errors are estimates calculated from a ± 5% increase in the FOM. See caption of Table S 5 for explanation of the parameters in the table.

Parameter	Si1	Si2	Parameter	Au2
d_{SiO2} (Å)	28.1 ± 1.9	12 ± 3	d_{SiO2} (Å)	19 ± 2
σ_{SiO2} (Å)	3 ± 2	3 ± 12	σ_{SiO2} (Å)	7.5 ± 0.7
ϕ_{SiO2}	0.85 ± 0.01	0.54 ± 0.12	d_{Ti} (Å)	14.6 ± 0.8
			d_{Au} (Å)	145.8 ± 0.8
d_{Silane} (Å)	15.0 ± 1.5	43 ± 4	d_{Thiol} (Å)	14.0 ± 1.2
ρ_{Silane} ($\times 10^{-6}$ Å^{-2})	2.56 ± 0.11	3.11 ± 0.16		
ϕ_{Silane}	0.93 ± 0.04	0.87 ± 0.08	ϕ_{Thiol}	1.00 ± 0.05
σ_{Silane} (Å)	22 ± 3	10 ± 8	σ_{Thiol} (Å)	3.8 ± 1.2
d_1 (Å)	121 ±16	100 ± 30	d_1 (Å)	180 ± 11
ρ_1 ($\times 10^{-6}$ Å^{-2})	4.93 ± 0.09	4.31 ± 0.11	ρ_1 ($\times 10^{-6}$ Å^{-2})	1.94 ± 0.09
ϕ_1	0.46 ± 0.01	0.59 ± 0.04	ϕ_1	0.86 ± 0.03
σ_1 (Å)	30 ± 40	90 ± 80	σ_1 (Å)	73 ± 9
d_2 (Å)	231 ± 19	340 ± 40	d_2 (Å)	155 ± 20
ρ_2 ($\times 10^{-6}$ Å^{-2})	5.37 ± 0.18	5.53 ± 0.18	ρ_2 ($\times 10^{-6}$ Å^{-2})	3.7 ± 0.2
ϕ_2	0.40 ± 0.02	0.44 ± 0.04	ϕ_2	0.51 ± 0.07
σ_2 (Å)	161 ± 7	200 ± 200	σ_2 (Å)	110 ± 30
d_3 (Å)	50 ± 6	300 ± 40	d_3 (Å)	280 ± 30
ρ_3 ($\times 10^{-6}$ Å^{-2})	5.53 ± 0.15	5.5 ± 0.8	ρ_3 ($\times 10^{-6}$ Å^{-2})	5.5 ±0.3
ϕ_3	0.82 ± 0.02	0.18 ± 0.08	ϕ_3	0.17 ± 0.04
σ_3 (Å)	158 ± 5	80 ± 40	σ_3 (Å)	50 ± 40
d_{dry1} (Å)	106 ± 7	277 ± 15	d_{dry1} (Å)	136 ±6
ρ_{dry1} ($\times 10^{-6}$ Å^{-2})	3.59 ± 0.13	4.07 ± 0.07	ρ_{dry1} ($\times 10^{-6}$ Å^{-2})	1.5 ± 0.1
σ_{dry1} (Å)	50 ± 20	89 ± 18	σ_{dry1} (Å)	59 ± 14
d_{dry2} (Å)	67 ± 7	129 ± 12	d_{dry2} (Å)	240 ± 10
ρ_{dry2} ($\times 10^{-6}$ Å^{-2})	4.14 ± 0.15	2.3 ± 0.4	ρ_{dry2} ($\times 10^{-6}$ Å^{-2})	3.5 ± 0.1
σ_{dry2} (Å)	25.1 ± 1.8	54 ± 10	σ_{dry2} (Å)	51 ± 9

Grafting density and the swelling of the layers

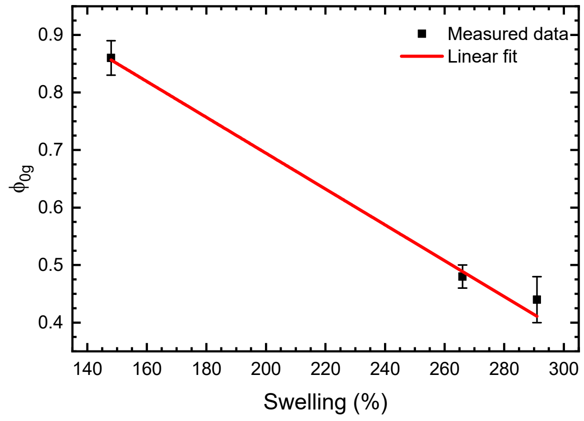

Figure S10. Comparison of the swelling and grafting densities on the measured data (dots) and a linear fit (line). The line was included merely to highlight the negative correlation, but the data is insufficient to state with any certainty whether the relation actually is linear.

Comparing polymer volume fraction profiles of different models

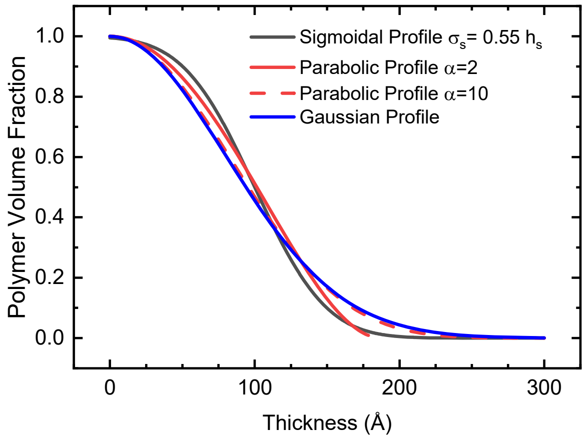

Figure S11. Different polymer volume fraction profiles, calculated from the same amount of polymer (corresponding to a 100 Å thick homogeneous slab).

Infrared spectroscopy for monitoring hydration

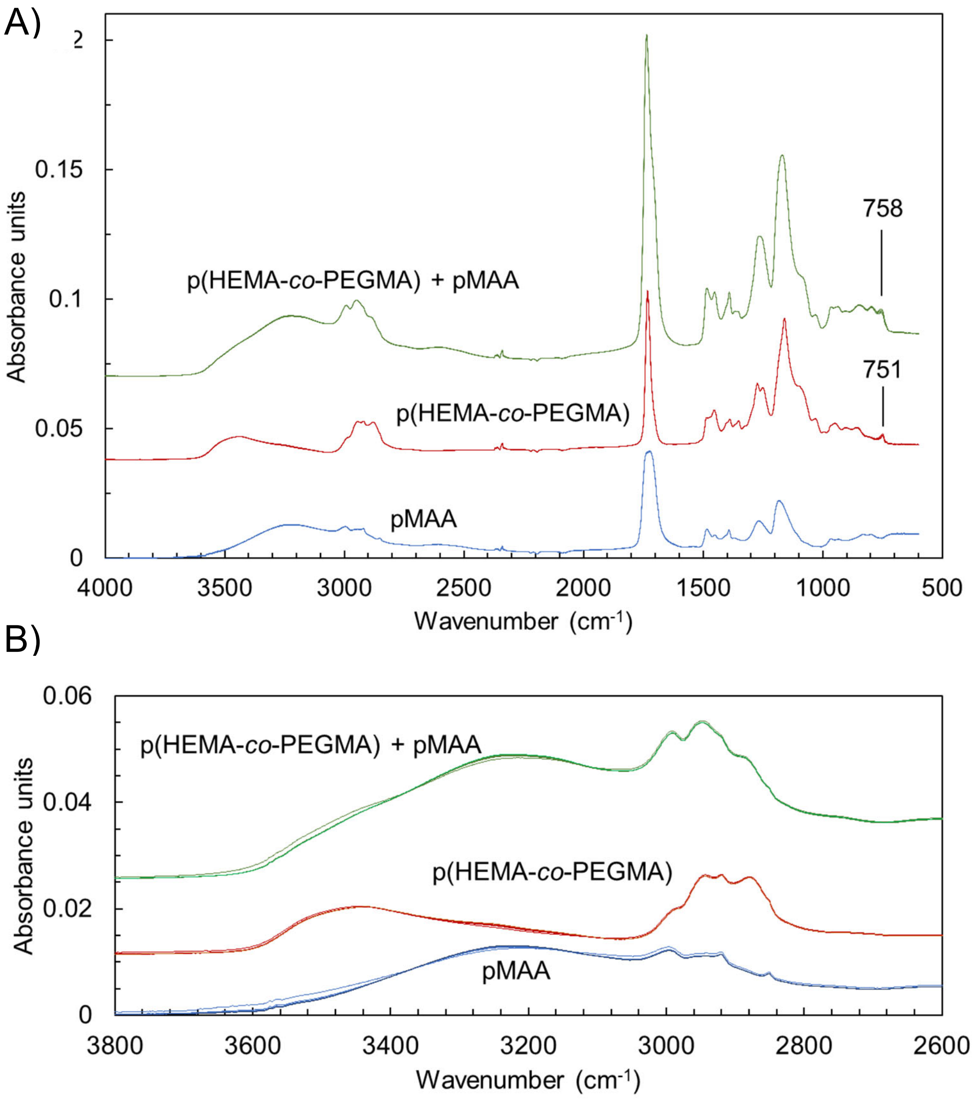

Figure S12. A) Infrared reflection-absorption spectra acquired on SI-PGP-prepared p(MAA) and p(HEMA-*co*-PEG$_{10}$MA) polymer films, and sequentially polymerized p(HEMA-*co*-PEG$_{10}$MA) + p(MAA) films. After soaking in water for ca 1 h, the samples were taken out of the water, quickly dried under a stream of N_2 gas until all visible water was removed, and then mounted in the N_2-purged sample chamber. Spectra in A) were obtained after 15 – 75 min, at 15 min intervals. B) Close-up of the data in A, showing, for each sample, also one *additional spectrum* whose acquisition finished ca 5 min after placing the sample in the purged chamber. In B), there are slight deviations between the first and the subsequent spectra in the O-H- stretching region (ca 3600-3100 cm^{-1}), but thereafter the only differences are very slight changes around 760-750 cm^{-1} for the poly(HEMA-*co*-PEG$_{10}$MA)-containing samples, as indicated in A). A number of C-H-deformation modes are expected in this region, and could indicate re-orientation of chain segments (as per the surface selection rule). However, overall, the O-H-bands in the 3500-3000 cm^{-1} range remain unchanged, as do also the carbonyl bands around 1700 cm^{-1} and ether C-O-C vibrations around 1200 cm^{-1}, which are all expected to be affected by hydration changes.

Appendix 1

```
import math
import numpy as np
import scipy.special as sp

Thick_Dry=100.0                                       #The dry thickness of the layer
Thick_Brush=500.0                                     #The hydrated thickness of the layer
Poly_VF=0.5                                           #The volume fraction at the interface
Exponent_Brush=0.0                                    #The value of the brush exponent
Interval=16.0          # 2^n, the maximum value the program will examine is 2^(n+1)
Precision=0.001                                       #The precision of the inversion

def Fx(X, a):                                         #Defining the function to be inverted
        return 2.0*a*sp.gamma(1.5+X)-sp.gamma(1+X)*np.sqrt(math.pi)

def Inverse(a, Fx, x0, eps):                          #Defining the bisection method
        x1=x0
        while abs(x0)>eps:
                x0=x0/2
                x1=x1-np.sign(Fx(x1, a))*x0
        return x1

Exponent_Brush=Inverse(Thick_Dry/(Thick_Brush*Poly_VF), Fx, Interval, Precision)
```

Appendix 2

```
import math
import numpy as np
import scipy.special as sp

Thick_Dry=100.0                                       #The dry thickness of the layer
Thick_Brush=0.0                                       #The hydrated thickness of the layer
Poly_VF=0.5                                           #The volume fraction at the interface
Roughness_Brush=100.0                                 #The value of the brush roughness
Interval=512.0         # 2^n, the maximum value the program will examine is 2^(n+1)
Precision=0.001                                       #The precision of the inversion

def Fx(X, a, b):                                      #Defining the function to be inverted
        return X*sp.erf(X/b)+X+b*np.exp(-(X/b)**2.0)/np.sqrt(np.pi)-a

def Inverse(a, b, Fx, x0, eps):                       #Defining the bisection method
        x1=x0
        while abs(x0)>eps:
                x0=x0/2
                x1=x1-np.sign(Fx(x1, a, b))*x0
        return x1

Thick_Brush=Inverse(2*Thick_Dry/Poly_VF, Roughness_Brush, Fx, Interval, Precision)
```
